# Association of miR-144 levels in the peripheral blood with COVID-19 severity and mortality

**DOI:** 10.1038/s41598-022-23922-2

**Published:** 2022-11-21

**Authors:** Alisia Madè, Simona Greco, Melanie Vausort, Marios Miliotis, Eric Schordan, Shounak Baksi, Lu Zhang, Ekaterina Baryshnikova, Marco Ranucci, Rosanna Cardani, Guy Fagherazzi, Markus Ollert, Spyros Tastsoglou, Giannis Vatsellas, Artemis Hatzigeorgiou, Hüseyin Firat, Yvan Devaux, Fabio Martelli

**Affiliations:** 1 Molecular Cardiology Laboratory, IRCCS Policlinico San Donato, Via Morandi 30, 20097 San Donato Milanese, MI Italy; 2 Cardiovascular Research Unit, Department of Precision Health, Luxembourg Institute of Health, 1445 Strassen, Luxembourg; 3 Hellenic Pasteur Institute, 11521 Athens, Greece; 4 DIANA-Lab, Department of Computer Science and Biomedical Informatics, University of Thessaly, 35131 Lamia, Greece; 5 Firalis SA, 35 Rue du Fort, 68330 Huningue, France; 6 Bioinformatics Platform, Luxembourg Institute of Health, L-1445 Strassen, Luxembourg; 7 Department of Cardiovascular Anesthesia and ICU, IRCCS Policlinico San Donato, Via Morandi 30, 20097 San Donato Milanese, MI Italy; 8 BioCor Biobank, UOC SMEL-1 of Clinical Pathology, Department of Pathology and Laboratory Medicine, IRCCS-Policlinico San Donato, Via Morandi 30, 20097 San Donato Milanese, MI Italy; 9 Deep Digital Phenotyping Research Unit, Department of Precision Health, Luxembourg Institute of Health, 1 A-B Rue Thomas Edison, 1445 Strassen, Luxembourg; 10 Department of Infection and Immunity, Luxembourg Institute of Health, 29, Rue Henri Koch, 4354 Esch-Sur-Alzette, Luxembourg; 11 Department of Dermatology and Allergy Center, Odense Research Center for Anaphylaxis (ORCA), University of Southern Denmark, 5000 Odense, Denmark; 12 Greek Genome Center, Biomedical Research Foundation, Academy of Athens, 11527 Athens, Greece

**Keywords:** miRNAs, SARS-CoV-2

## Abstract

Coronavirus disease-2019 (COVID-19) can be asymptomatic or lead to a wide symptom spectrum, including multi-organ damage and death. Here, we explored the potential of microRNAs in delineating patient condition and predicting clinical outcome. Plasma microRNA profiling of hospitalized COVID-19 patients showed that miR-144-3p was dynamically regulated in response to COVID-19. Thus, we further investigated the biomarker potential of miR-144-3p measured at admission in 179 COVID-19 patients and 29 healthy controls recruited in three centers. In hospitalized patients, circulating miR-144-3p levels discriminated between non-critical and critical illness (AUC_miR-144-3p_ = 0.71; *p *= 0.0006), acting also as mortality predictor (AUC_miR-144-3p_ = 0.67; *p *= 0.004). In non-hospitalized patients, plasma miR-144-3p levels discriminated mild from moderate disease (AUC_miR-144-3p_ = 0.67; *p *= 0.03). Uncontrolled release of pro-inflammatory cytokines can lead to clinical deterioration. Thus, we explored the added value of a miR-144/cytokine combined analysis in the assessment of hospitalized COVID-19 patients. A miR-144-3p/Epidermal Growth Factor (EGF) combined score discriminated between non-critical and critical hospitalized patients (AUC_miR-144-3p/EGF_ = 0.81; *p *< 0.0001); moreover, a miR-144-3p/Interleukin-10 (IL-10) score discriminated survivors from nonsurvivors (AUC_miR-144-3p/IL-10_ = 0.83; *p *< 0.0001). In conclusion, circulating miR-144-3p, possibly in combination with IL-10 or EGF, emerges as a noninvasive tool for early risk-based stratification and mortality prediction in COVID-19.

## Introduction

Coronavirus disease 2019 (COVID-19) is caused by the infection of the single-stranded RNA virus SARS-CoV-2. Infection can be asymptomatic or may cause a wide spectrum of symptoms, from mild upper respiratory tract involvement to severe hypoxia with acute respiratory distress syndrome, life-threatening sepsis, coagulation dysfunction, and multi-organ involvement, including kidney, nervous system and cardiovascular damage^1–4^. COVID-19 can result in systemic hyper-activation of the inflammatory system caused by the release of large amounts of pro-inflammatory cytokines (also named “cytokine storm”) which can lead to rapid clinical deterioration^5,6^. Indeed, 20–30% of hospitalized COVID-19 patients develop a severe form of the disease requiring intensive care unit (ICU) admission with high death rates^7^.

Currently available vaccines have been demonstrated to be highly effective in protecting from severe COVID-19 forms^8–13^. However, a still large portion of the global population remains insufficiently protected for a variety of reasons^14^. Moreover, immunity, whether infection- or vaccination-derived, will wane, creating opportunities for continued SARS-CoV-2 transmission^15^. Thus, there is need for dependable tools to delineate patient condition and predict clinical outcomes. Although risk assessment based on clinical characteristics and classical biomarkers has been advanced significantly, early identification of severe patients requiring ICU admission or at risk of death is still highly challenging^16^. The investigation of new biomarkers provided by emerging approaches such as transcriptomics, may facilitate the identification of novel tools for more effective management of COVID-19 patients^17^.

microRNAs (miRNAs) are small non-coding RNAs (9–25 nt) that decrease target mRNA stability and/or repress their translation^18^. Interestingly, miRNAs are also readily detectable in the peripheral blood fluids, such as plasma and serum, and have been investigated as diagnostic and prognostic biomarkers for a variety of diseases, including myocardial infarction, cancer, sepsis and viral infections^19–23^. While protein biomarkers for COVID-19 have been extensively studied^24–27^, only few studies have explored the potential of circulating miRNAs as biomarkers, as well as that of combined miRNA/protein signatures^17,28,29^.

In this study, we investigated the circulating miRNA profile of hospitalized COVID-19 patients and assessed their potential role as biomarkers of disease severity and mortality in a broad spectrum of disease manifestations recruiting patients from three centers across Europe. The potential of a combined use of miRNAs and inflammatory cytokines was also explored.

## Results

### miR-144-3p levels are dynamically regulated in the plasma of COVID-19 patients

In a small pilot study, RNA sequencing was performed on platelet-poor plasma samples derived from the peripheral blood of 3 surviving and 8 nonsurviving COVID-19 patients, sex- and age-matched recruited at PSD hospital. For each patient, samples collected on hospital admission (T_0_) and before discharge or death (T_1_) were compared. By analyzing the miRNA-sequencing profile of surviving patients on their way to recovery and nonsurviving patients, in which the T_1_ sample was harvested during the most severe phase of the disease, we aimed to identify miRNAs that were dynamically modulated during the disease course of each patient.

Out of 734 miRNAs detected, miR-144-3p and miR-5582-3p were differentially expressed (*p *< 0.001) between T_0_ and T_1_ sampling in surviving, but not in nonsurviving patients (Supplementary Fig. 1a-b).

**Figure 1:**
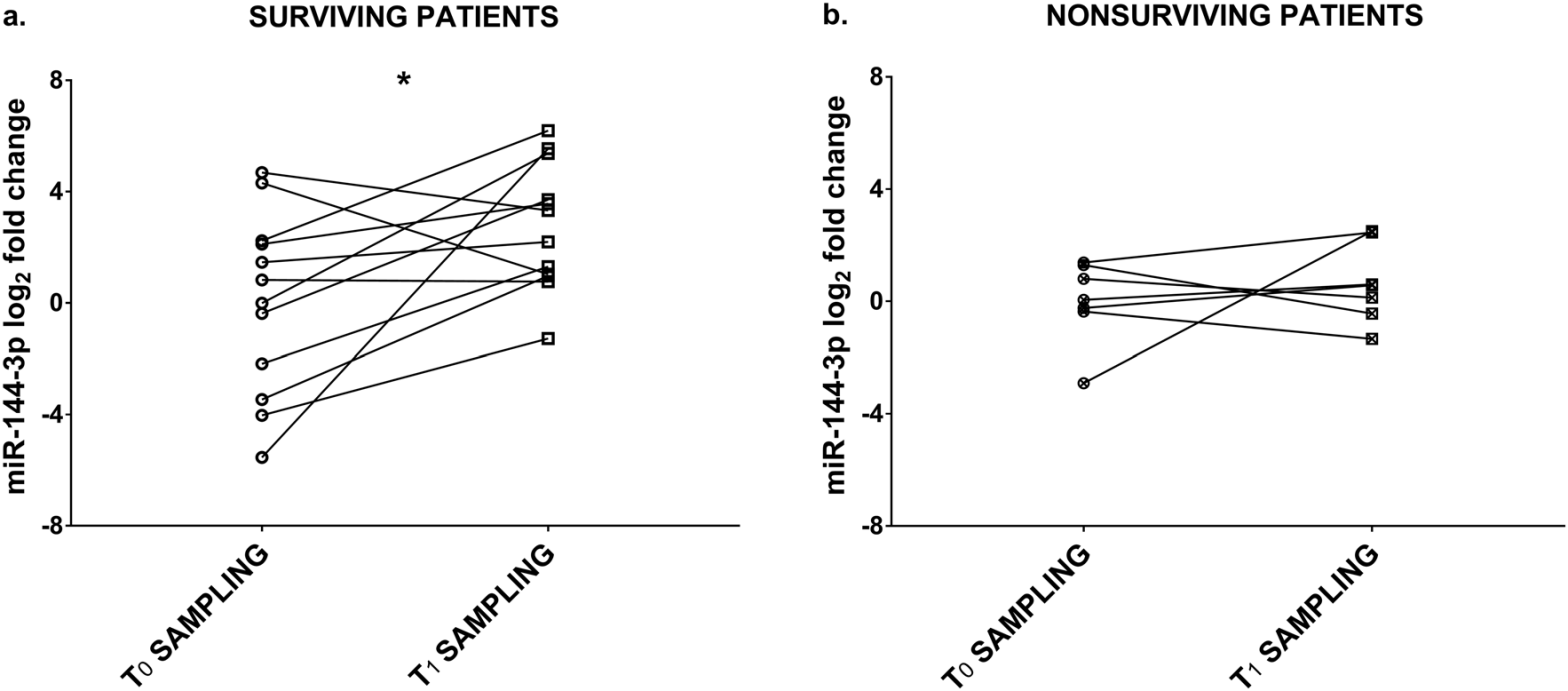
Changes in miR-144-3p levels between T_0_ and T_1_ sampling in critical COVID-19 patients. Patients analyzed were ICU-admitted subjects requiring endotracheal intubation. miR-144-3p levels were measured by qPCR in T_0_ (admission) and T_1_ (discharge or death) plasma samples of each patient. Values are expressed as log_2_ fold change compared to the average values of the first sampling for each group. miR-144-3p expression levels were rescued over time in surviving (**a**) but not in nonsurviving COVID-19 patients (**b**). Mann–Whitney test (two groups) was performed for statistical comparison. Survivors n = 12; nonsurvivors n = 7; *p < 0.05.

The top scoring miR-144-3p was selected for validation by qPCR in a group (n = 19) of surviving and nonsurviving COVID-19 patients, sex- and age-matched. All patients were admitted to the ICU, indicating similar disease severity at T_0_ on admission. To minimize the possible interfering effect of heparin anticoagulant therapy during hospitalization, RNA extracted from plasma was treated with heparinase 1 before measurement. Figure 1 shows that, while miR-144-3p levels were similar at T_0_ and T_1_ sampling in nonsurviving patients, they increased significantly at discharge in survivors.

These experiments, although conducted in a limited number of patients, indicate that miR-144-3p plasma level is dynamically regulated in response to COVID-19, prompting a further investigation of its potential as a disease biomarker in COVID-19.

### miR-144-3p and -5p levels accurately discriminate COVID-19 patients from healthy controls

**Figure 2:**
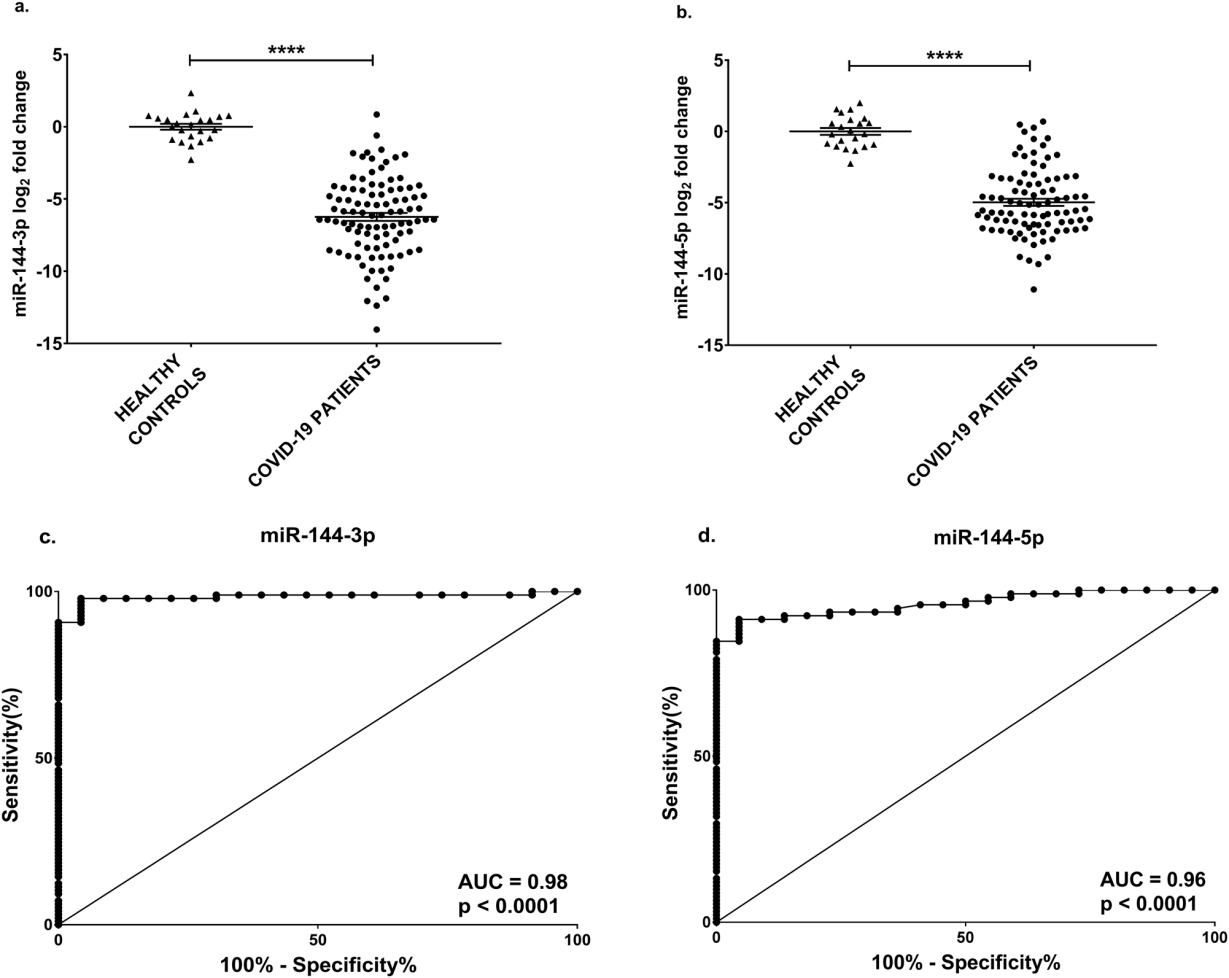
miR-144 levels are decreased in hospitalized COVID-19 patients and discriminate patients from healthy controls. miR-144-3p (**a**) and miR-144-5p (**b**) plasma levels were measured by qPCR. Values are expressed as log_2_ fold change compared to controls and shown as dot- plots indicating mean ± SEM. Unpaired t-test (two groups) was used for statistical comparison. The ROC curves show the sensitivity and specificity of miR-144-3p (**c**) and miR-144-5p (**d**) to distinguish COVID-19 patients hospitalized at PSD from healthy controls. Controls n = 23; COVID-19 patients n = 97; ****p ≤ 0.0001.

It was tested whether the levels of plasma miR-144-3p were modulated in a group of 97 hospitalized COVID-19 patients recruited at PSD. Patients were sampled on admission and displayed different severity degrees and outcomes (Table 1). They were compared against sex- and age-matched controls that had never suffered from COVID-19 before blood harvesting. Measurement by qPCR showed that miR-144-3p was readily detectable in all plasma samples and that miR-144-3p levels were significantly lower in COVID-19 patients compared to controls (Fig. 2a).

**Table 1:** Characteristics of the COVID-19 patients hospitalized at PSD.

	COVID-19 PATIENTS				
	All (n = 97)	Severity 1 † (n = 3)	Severity 2 ‡ (n = 15)	Severity 3 § (n = 30)	Severity 4 ¶ (n = 49)
Age, median (range)	67 (26–86)	67 (58–78)	66 (26–85)	68 (45–85)	68 (45–86)
Gender, male, n (%)	63 (63)	1 (33)	9 (60)	19 (63)	34 (69)
Outcome, dead patients (%)	51 (53)	0 (0)	1 (7)	15 (50)	35 (71)
Days of hospitalization, median	10	5	7	14	8
**Medical history/comorbidities, n (%)**					
Smoking					
Current smoker	5 (5)	0 (0)	1 (7)	2 (7)	2 (4)
Former smoker	7 (7)	0 (0)	2 (13)	1 (3)	4 (8)
Hypertension	56 (58)	2 (67)	7 (45)	19 (63)	28 (57)
Diabetes	25 (26)	1 (33)	1 (7)	8 (27)	15 (31)
Obesity	22 (23)	1 (33)	2 (13)	6 (20)	13 (27)
Ischemic cardiomyopathy	23 (24)	0 (0)	3 (20)	8 (27)	12 (24)
Chronic heart failure	9 (9)	0 (0)	1 (7)	7 (23)	1 (2)
Atrial fibrillation	9 (9)	0 (0)	3 (20)	5 (17)	1 (2)
Left ventricular dysfunction	3 (3)	0 (0)	1 (7)	2 (7)	0 (0)
Congenital heart diseases	0 (0)	0 (0)	0 (0)	0 (0)	0 (0)
Chronic obstructive pulmonary disease	8 (8)	0 (0)	1 (7)	3 (10)	4 (8)
Asthma	5 (5)	0 (0)	2 (13)	1 (3)	2 (4)
Cancer	3 (3)	0 (0)	1 (7)	2 (7)	0 (0)
Pre-existing stroke	5 (5)	0 (0)	0 (0)	2 (7)	3 (6)
Chronic neurological disorders	3 (3)	0 (0)	1 (7)	1 (3)	1 (2)
Chronic kidney disorders	6 (6)	0 (0)	1 (7)	3 (10)	2 (4)
Liver disorders	2 (2)	0 (0)	1 (7)	1 (3)	0 (0)
Chronic gut inflammation	1 (1)	0 (0)	1 (7)	0 (0)	0 (0)
Other disorders	41 (42)	3 (100)	8 (53)	13 (43)	17 (35)
**Hospital therapy, n (%)**					
Antiviral drug	24 (25)	0 (0)	1 (7)	4 (13)	19 (39)
Cortisone therapy	78 (80)	2 (67)	13 (87)	23 (77)	40 (82)
Immunosuppressive therapy	4 (4)	0 (0)	0 (0)	1 (3)	3 (6)
Muscle relaxant therapy	47 (48)	0 (0)	0 (0)	3 (10)	44 (90)
Amine therapy	24 (25)	0 (0)	0 (0)	2 (7)	22 (45)
Sedative	53 (55)	0 (0)	1 (7)	6 (20)	46 (94)
Hydroxychloroquine/chloroquine	30 (31)	1 (33)	2 (13)	9 (30)	18 (37)
Antibiotic therapy	90 (93)	3 (100)	12 (80)	29 (97)	46 (94)
Therapy with NSAID	13 (13)	0 (0)	2 (13)	3 (10)	8 (16)
Antifungal therapy	4 (4)	0 (0)	1 (7)	0 (0)	3 (6)
Anticoagulant therapy	91 (94)	3 (100)	13 (87)	28 (93)	47 (96)

^†^Severity 1: patients not requiring oxygen therapy; ‡ Severity 2: patients requiring oxygen therapy; § Severity 3: patients requiring CPAP therapy; ¶ Severity 4: ICU-admitted patients with endotracheal intubation*.*

The potential inhibitory effect of unreported heparin treatment was assessed on a subgroup of 13 samples. Assessment of miR-144-3p levels in RNA samples with or without heparinase 1 treatment, yielded no significant differences (not shown). Moreover, no significant differences were observed in miR-144-3p plasma levels between male and female COVID-19 patients (Supplementary Fig. 2a). Indeed, multivariate analysis indicated that sex and age variables did not have a significant correlation with miR-144-3p levels.

Functional mature miRNAs are processed from one or both arms of an hairpin precursor, and are named -3p and -5p according to the originating arm (3’ and 5’, respectively)^18^. miRNAs originating from the same precursor may or may not be co-regulated and display similar patterns of expression in the plasma^20^. Thus, we tested whether miR-144-5p was also detectable in the plasma and if its levels were dysregulated in COVID-19 patients. qPCR measurement showed that miR-144-5p was readily detectable in all plasma samples and that its levels were significantly lower in COVID-19 patients compared to controls (Fig. 2b). Again in this case, no significant differences in miR-144-5p levels were observed between male and female COVID-19 patients (Supplementary Fig. 2b).

Interestingly, miR-144-3p and -5p levels displayed significant negative correlations with clinically relevant parameters in COVID-19 patients, such as C-reactive protein (CRP) concentration, platelet-lymphocyte ratio, and neutrophil–lymphocyte ratio (Supplementary Fig. 3)^1^. miR-144-3p expression is strongly induced upon differentiation of erythroid cells^30,31^. Nevertheless, no significant correlations with hematocrit or red blood cells levels were observed.

To assess the potential of miR-144-3p and -5p as biomarkers of COVID-19, ROC curves were calculated. ROC analysis indicated that both miR-144-3p and miR-144-5p levels individually discriminated hospitalized COVID-19 patients from healthy controls with high efficiency, displaying an AUC of 0.98 and 0.96, respectively (Fig. 2c-d).

In conclusion, the levels of both miR-144-3p and -5p were decreased in hospitalized COVID-19 patients and discriminated COVID-19 patients from healthy controls.

### Association between circulating miR-144-3p/-5p and COVID-19 severity in hospitalized patients

The identification of biomarkers associated to COVID-19 severity and outcome in hospitalized patients is considered particularly valuable for the clinical management of the patients^1,3,17^.

**Figure 3:**
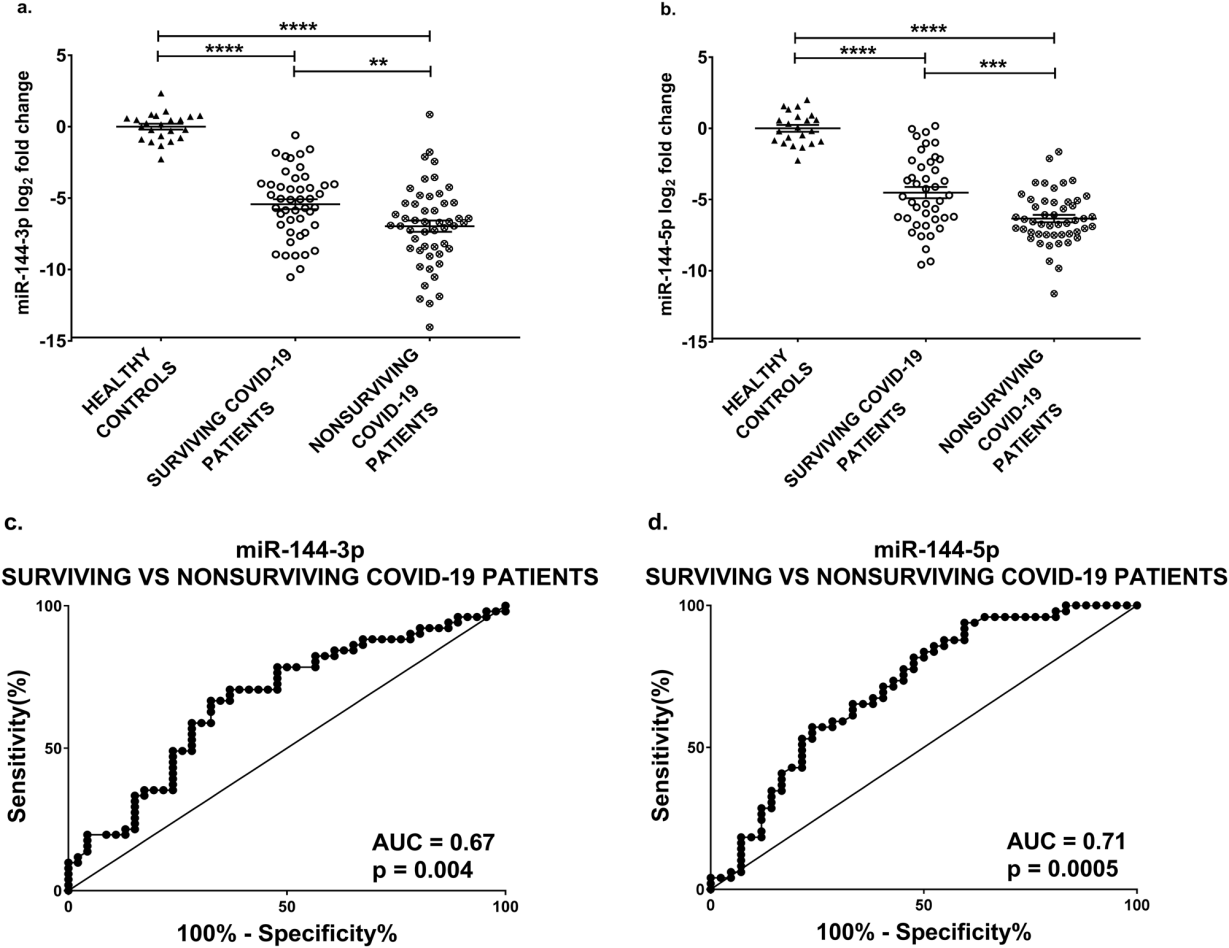
miR-144 levels are decreased in nonsurviving COVID-19 patients and discriminate nonsurviving patients from survivors. miR-144-3p (**a**) and miR-144-5p (**b**) plasma levels were measured by qPCR in COVID-19 patients hospitalized at PSD and healthy controls. Values are expressed as log_2_ fold change compared to controls and shown as dot-plots indicating mean ± SEM. Both miR-144-3p and miR-144-5p expression levels were lower in nonsurviving patients compared to survivors. ANOVA test, followed by Tukey’s post-hoc test, was performed for statistical comparison. The ROC curves show the sensitivity and specificity of miR-144-3p (**c**) and miR-144-5p (**d**) to distinguish surviving from nonsurviving COVID-19 patients. Controls n = 23; survivors n = 42–46; nonsurvivors n = 49–51; **p ≤ 0.01; ***p ≤ 0.001; ****p ≤ 0.0001.

To this aim, PSD COVID-19 patients were classified according to a positive (alive at discharge) or negative (death) outcome (Table 1). Figure 3a-b shows that plasma levels of miR-144-3p and -5p on admission not only decreased in both COVID-19 groups compared to healthy controls, but also in nonsurviving compared to surviving patients. Accordingly, ROC curve analysis indicated that miR-144-3p and -5p levels allowed a statistically significant discrimination between the latter groups, with AUC values of 0.67 and 0.71, respectively (Fig. 3c-d).

**Figure 4:**
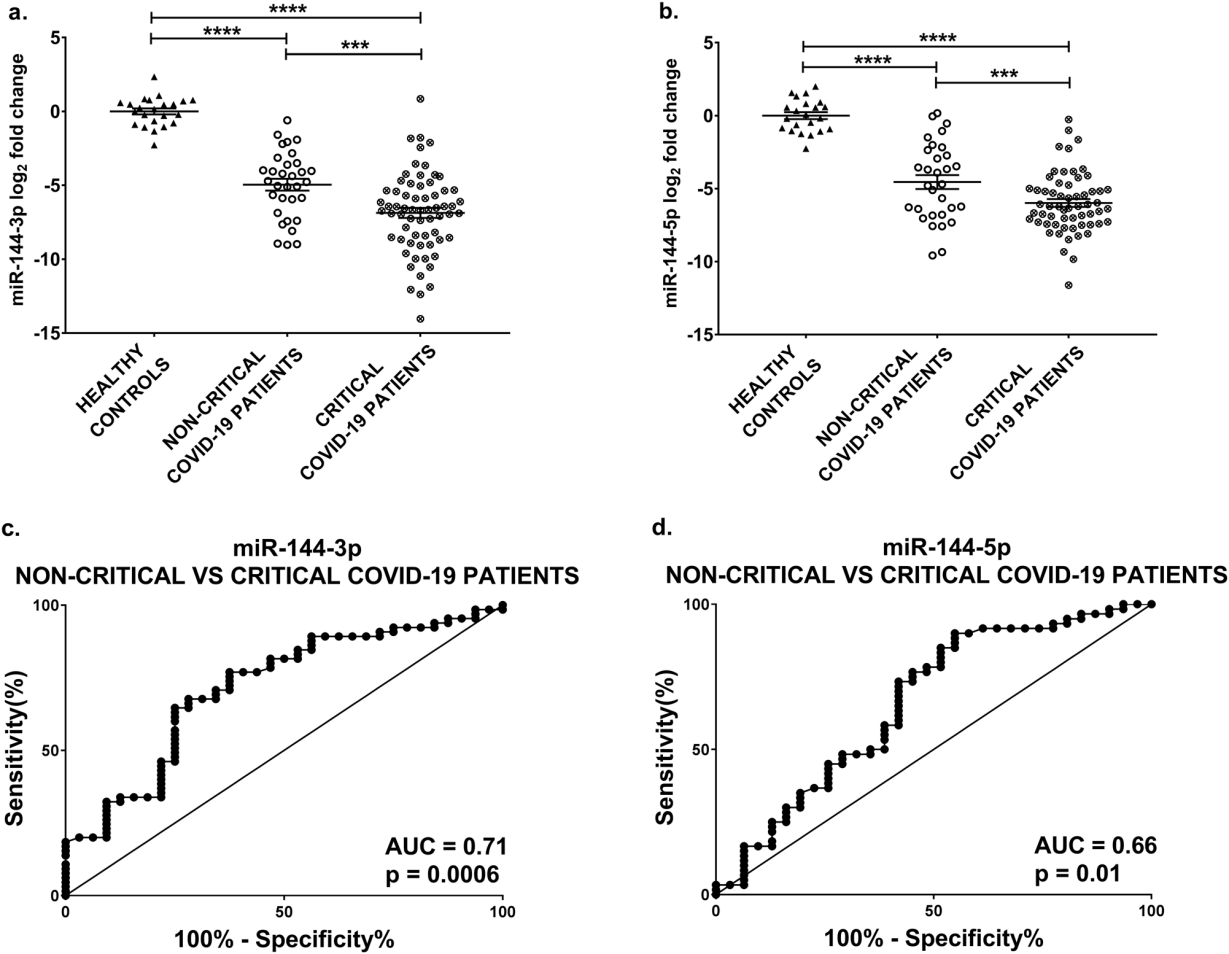
miR-144 levels are decreased in critical COVID-19 patients and discriminate critical from non-critical hospitalized patients. miR-144-3p (**a**) and miR-144-5p (**b**) plasma levels were measured by qPCR in hospitalized patients at PSD and healthy controls. Values are expressed as log_2_ fold change compared to controls and shown as dot-plots indicating mean ± SEM. Both miR-144-3p and miR-144-5p expression levels decreased in critical compared to non-critical patients. ANOVA test, followed by Tukey’s post-hoc test, was performed for statistical comparison. The ROC curves show the sensitivity and specificity of miR-144-3p (**c**) and miR-144-5p (**d**) to distinguish non-critical from critical COVID-19 patients. Controls n = 23; non-critical patients n = 31–32; critical patients n = 60–65; ***p ≤ 0.001; ****p ≤ 0.0001.

A particularly relevant element for the clinical course of COVID-19 patients, other than death, is the need for ICU admission and endotracheal intubation. Thus, hospitalized COVID-19 patients (Table 1) were further classified in critical (class 4 or nonsurvivors) and non-critical (class 1, not requiring oxygen therapy, classes 2 and 3, requiring oxygen therapy or CPAP, respectively, without a lethal outcome). Differences in sex and age composition between these COVID-19 groups and a control group constituted by healthy subjects were not statistically significant. Figure 4a-b shows that plasma miR-144-3p and -5p levels on admission were lower in critical compared to non-critical hospitalized patients. ROC curve analysis indicated that miR-144-3p and -5p levels enabled a statistically significant discrimination between non-critical and critical patients, with AUC values of 0.71 and 0.66, respectively (Fig. 4c-d).

A possible source of plasma miR-144-3p are blood cells. Thus, we took advantage of a subset of COVID-19 patients for which PBMCs were harvested on admission in parallel with plasma samples. miR-144-3p PBMC levels in 15 non-critical and 15 critical hospitalized COVID-19 patients were compared to those measured in 8 healthy controls. Supplementary Fig. 4a shows that miR-144-3p levels in PBMCs were significantly increased in both COVID-19 patient groups compared to controls, indicating an opposite modulation to that observed in plasma samples. However, no significant difference in PBMC miR-144-3p levels was observed between non-critical and critical patients, indicating that plasma miR-144-3p levels are not simply an opposite correlation of those present in the PBMCs. Moreover, when the levels of the precursor transcript of miR-144 were measured (Supplementary Fig. 4b), no significant differences were observed in COVID-19 patients compared to controls, indicating that the increase of the mature levels of miR-144-3p is due to post-transcriptional regulations.

In order to further validate the association between circulating miR-144 and COVID-19 severity, a small group of patients recruited in a different European center, UGHL, were analyzed. Six critical patients admitted at ICU were compared to 6 sex-matched healthy volunteers. Clinical and demographic data are shown in Supplementary Table 1. The levels of miR-144-3p and -5p were measured in serum samples by small RNA-sequencing: supplementary Fig. 5 shows that both miR-144-3p and -5p levels were significantly lower in ICU patients compared to controls.

### Association between circulating miR-144-3p levels and COVID-19 severity in non-hospitalized patients

**Figure 5:**
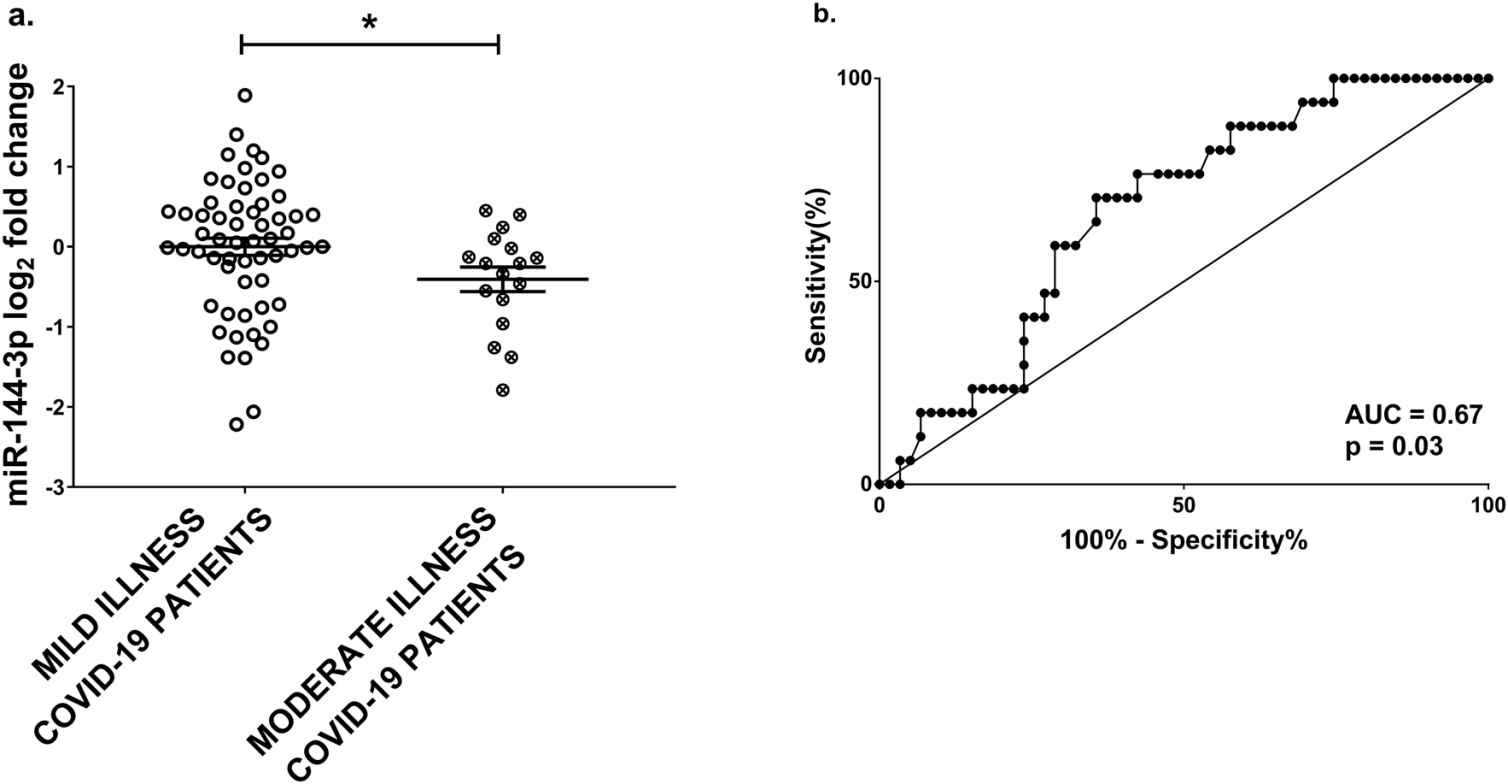
miR-144-3p in mild and moderate COVID-19 non-hospitalized patients recruited at LIH. miR-144-3p plasma levels were measured by qPCR. Values are expressed as log_2_ fold change compared to mild illness patients and shown as dot- plots indicating mean ± SEM. miR-144-3p expression levels are lower in more ill patients. Mann–Whitney test (two groups) was performed for statistical comparison (**a**). The ROC curve shows the sensitivity and specificity of miR-144-3p to distinguish mild from moderate COVID-19 patients (**b**). Mild illness patients n = 59; moderate illness patients n = 17; *p < 0.05.

We additionally studied the association of miR-144-3p and -5p plasma abundance in patients displaying less severe symptoms and not requiring hospitalization. For this purpose, 76 participants, positively tested for SARS-CoV-2, were recruited at LIH and classified according to NIH disease-severity scale^32^ into mildly or moderately ill. According to this classification, patients with mild illness may exhibit a variety of symptoms such as fever, cough, sore throat, malaise, headache, muscle pain, nausea, vomiting, diarrhea, loss of taste and smell, without shortness of breath, dyspnea or abnormal imaging. In addition, patients with moderate illness also showed evidence of lower respiratory disease during clinical assessment or imaging, but were not hospitalized according to their blood oxygen saturation ≥ 94%^32^. The demographic and clinical data are shown in Supplementary Table 2. Circulating levels of miR-144-3p and -5p were measured in plasma samples by qPCR showing that both miR-144 mature forms were detectable in all plasma samples. Interestingly, while miR-144-3p displayed lower levels in moderately ill patients (Fig. 5a), the levels of miR-144-5p were not significantly changed (Supplementary Fig. 6a), indicating that the two miR-144 mature forms were not co-regulated in this class of patients. ROC curve analysis shows that miR-144-3p significantly discriminated moderate from mild illness in COVID-19 patients (Fig. 5b).

The genomic localization of miR-451a is only 180 bp away from the mir-144 locus and the two precursors are co-expressed into a bi-cistronic primary transcript^33^. Thus, it was tested whether miR-451a levels were affected in the plasma of non-hospitalized patients. Supplementary Fig. 6b shows that no significant changes were observed.

### miR-144/cytokine combined analysis in COVID-19 hospitalized patients

Cytokine storm is a major cause of damage in the lung and other tissues of COVID-19 patients, causing the uncontrolled recruitment and activation of immune-inflammatory cells^5,6^. Accordingly, pro-inflammatory cytokines are considered valuable biomarkers of systemic inflammation.

Thus, we explored the potential functional associations of predicted targets of miR-144-3p and -5p using the Targetscan tool^34^. Interestingly, enriched pathways included terms involving cytokine and growth factor response-pathways (e.g. Epidermal Growth Factor Receptor and TGF-beta signaling pathways) consistent with the systemic inflammation characterizing severe COVID-19 disease (Supplementary Fig. 7).

These observations prompted us to take advantage of an independent serum proteomic analysis conducted in PSD hospitalized COVID-19 patients (Greco & Martelli, unpublished); patient characteristics are summarized in Supplementary Table 3. In this study, a panel of 45 serum cytokines was measured and 31 of them passed technical quality checks. Among the serum samples analyzed in this unpublished study, 62 were harvested in parallel with the plasma samples used for miR-144 measurement and allowed a combined miR-144/cytokine analysis.

Correlation analysis showed a negative correlation between miR-144-3p/-5p and several inflammatory cytokine levels, including IL-8 and IL-10^35,36^ (r = − 0.6 and − 0.4 with miR-144-5p, respectively), indicating higher pro-inflammatory cytokines levels in patients displaying lower miR-144-3p/-5p levels (Supplementary Fig. 8).

Given the TGF-β-induced chronic immune response observed in severe COVID-19 patients^37^ and prompted by the functional associations of miR-144 predicted targets with TGF-beta signaling pathways, we also measured plasma TGF-β levels that was not included in the cytokine panel used for the seroproteomic analysis. Measuring TGF-β1 values in 22 non-critical and 36 critical hospitalized COVID-19 patients, the observed average values were 50.0 ± 7.9 pg/mL and 95.8 ± 18.9 pg/mL respectively; this difference was not statistically significant (*p *= 0.07), possibly due to the high variability of TGF-β1 levels in critical patients and to the limited patient numerosity.

Overall, these observations stimulated us to explore the added value of a miR-144/cytokine combined analysis in discriminating COVID-19 patients of different severity.

**Figure 6:**
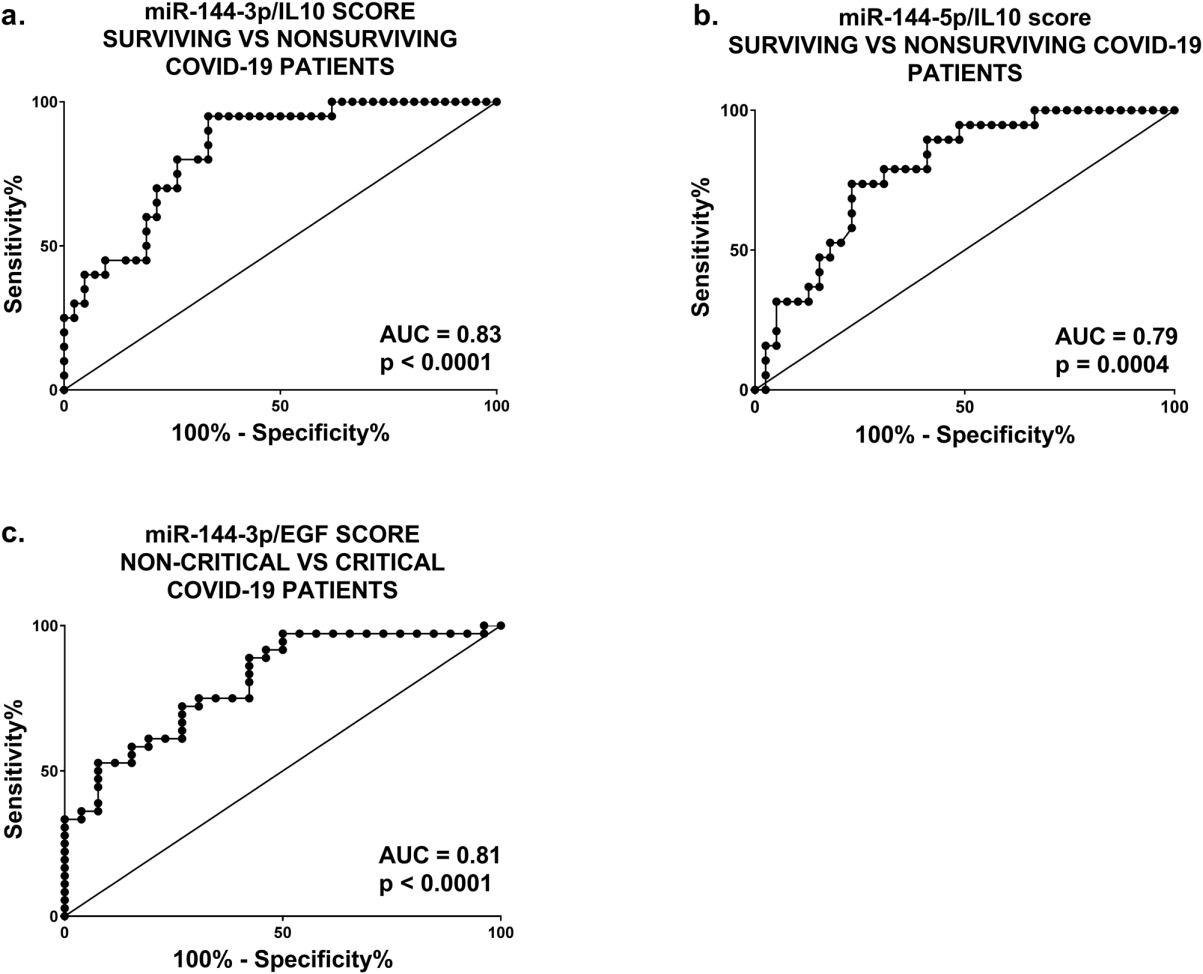
Discriminating potential of miR-144/cytokine combined analysis in COVID-19 hospitalized patients. The ROC curves show the sensitivity and specificity of miR-144-3p/IL-10 (**a**) and miR-144-5p/IL-10 (**b**) scores to distinguish surviving from nonsurviving COVID-19 patients. Survivors n = 42; nonsurvivors n = 20. In panel (**c**), the ROC curve shows the sensitivity and specificity of miR-144-3p/EGF score to distinguish non-critical from critical COVID-19 patients. Non-critical patients n = 26; critical patients n = 36.

Comparing surviving *vs.* nonsurviving COVID-19 patients, after multiple comparison adjustment, IL-10 levels were significantly (adj. *p *< 0.01) higher in nonsurviving patients (434 ± 68.51 pg/mL) compared to survivors (195 ± 25.66 pg/mL). Interestingly, combined miR-144-3p/IL-10 and miR-144-5p/IL-10 scores allowed a significant discrimination between surviving and nonsurviving patients, with AUC values of 0.83 and 0.79, respectively (Fig. 6a-b). These AUC values are higher than those obtained by individual analysis of miR-144-3p or -5p (Fig. 3), as well as of IL-10 alone (AUC_IL-10_ = 0.77, *p *= 0.0006).

On the other hand, comparing non-critical *vs*. critical COVID-19 patients, EGF levels were found to be significantly (adj. *p *< 0.005) lower in critical (258 ± 25.99 pg/mL) compared to non-critical (452 ± 43.67 pg/mL) patients. The combined miR-144-3p/EGF score allowed a significant discrimination between non-critical and critical patients, with an AUC_miR-144-3p/EGF_ value of 0.81 (Fig. 6c), improving the AUC obtained analyzing EGF alone (AUC_EGF_ = 0.77, *p *= 0.004). Conversely, no increased discrimination power was observed combining miR-144-5p and EGF values.

We also tested the levels of IL-10 and EGF mRNAs in PBMCs of COVID-19 patients and controls, but none significant differences were observed (Supplementary Fig. 9a–b).

Overall, these data support the use of a combined miRNA/cytokine analysis to identify predictors of severity and mortality.

## Discussion

The identification of noninvasive and flexible indicators assisting the clinical management of COVID-19 patients is a still unmet need. Indeed, while available vaccines have demonstrated a good efficacy in protecting from severe COVID-19 forms and death^8–13^, still a large population remains insufficiently protected due to various reasons including health conditions, insufficient access to vaccines, or lack of trust in the safety of the vaccines^14^. Moreover, immunity, whether infection- or vaccination-derived, will wane, creating opportunities for continued SARS-CoV-2 transmission^15^ and it cannot be ruled out that currently available vaccines might have reduced efficacy against possible SARSs-CoV-2 variants that might emerge in the future.

On the other side, an increasing number of new therapies for COVID-19 are being developed and many have already been approved by the European Medicines Agency and/or the Food and Drugs Administration, such as corticoids, anti-SARS-CoV-2 antibodies, DARPin, and, more recently, other antiviral drugs, allowing to reduce mortality rates and ICU hospitalization burdens^32,38^. In this context, the identification of biomarkers that provide better stratification of SARS-CoV-2 infected patients being at risk to develop severe outcomes, is of paramount importance for personalized healthcare.

In this study, we investigated the still insufficiently explored field of miRNA dysregulation upon COVID-19^39–43^. By comparing the plasma miRNA profiles in a pilot group of patients, we identified miR-144-3p as dynamically altered according to disease status and outcomes. This prompted us to validate its value as a predictor of non-critical or critical disease and as a predictor of mortality in a broader study, encompassing three independent centers for a total of 179 COVID-19 patients and 29 controls, representing one of the largest circulating miRNA studies in COVID-19^41^.

The comparison of critically ill patients only to healthy controls could overestimate the performance of new biomarkers. Thus, we analyzed hospitalized patients with different severity (PSD and UGHL groups), as well as non-hospitalized patients displaying milder forms of the disease (LIH), providing a comprehensive view of COVID-19 pathophysiology.

Interestingly, miR-144 displayed an informative value in spite of differences of the matrix used, platelet-poor plasma, plasma or serum analyzed at PSD, LIH and UGHL, respectively. A variety of measurement techniques was adopted, ranging from targeted and non-targeted RNA-sequencing to different qPCR techniques, indicating the robustness of miR-144 as a potential COVID-19 marker.

The -3p and -5p forms of miR-144 displayed very similar patterns of expression in the plasma/serum of hospitalized patients (PSD and UGHL cohorts), allowing the discrimination of patients based on outcome or illness severity. On the other side, only miR-144-3p was dysregulated in less severe COVID-19 patients (LIH cohort). The reasons for this discrepancy are not known, but they may relate to important differences in the underlying disease, as miR-144-3p and -5p plasma levels may reflect the step-wise progression of COVID-19.

Previous studies also identified decreased levels of miR-144-3p and/or -5p in the peripheral blood of COVID-19 patients^29,41,44,45^. However, none of these investigations fully explored its potential as biomarker of mortality and severity; other differences are that some of these studies analyzed lower numbers of patients^44^ and whole blood instead of plasma/serum^45^.

A prominent role of miR-144 has been described in erythroid differentiation and homeostasis^30,31^. However, no significant correlations with hematocrit or red blood cells levels were observed in hospitalized COVID-19 patients. Moreover, a possible increased hemolysis in the peripheral blood of more severe patients should lead to increased levels of erythrocyte-enriched miRNA species, while we observed lower miR-144 levels in this group of patients, indicating this type of artifact as highly unlikely.

Also interesting is the modulation of miR-144-3p we found in PBMCs of COVID-19 patients, suggesting that miR-144-3p levels are extraordinarily sensitive to the disease. However, the opposite modulation observed in plasma and PBMCS, as well as the similar modulation in critical and non-critical patients, suggest that these modulations may reflect different aspects of the disease. The increase of mature miR-144-3p observed in PBMCs of COVID-19 patients was not due to increased transcription, as the levels of its precursor transcript were unaffected. Different mechanisms may explain these results, but it is tempting to speculate that, at least in part, lower release of miR-144-3p from the PBMCs of COVID-19 patients may be cause the decrease in the plasma levels^20,21^.

A direct interaction between miR-144 and SARS-CoV-2 has never been reported. However, ectopic expression of miR-144-3p in mouse lung epithelium facilitates the increased replication of influenza virus, encephalomyocarditis virus and vesicular stomatitis virus, altering the cellular antiviral transcriptional landscape^46^. Considering that the intra- and extra cellular levels of a miRNA often display opposite modulation patterns^20,21^, it is tempting to speculate that something similar may occur also in the lung epithelium of COVID-19 patients. Indeed, according to DIANA-miTED database, both miR-144-3p and -5p are highly expressed in human lung^47^.

Functional associations of miR-144-3p and -5p predicted targets included terms involving cytokine and growth factor response-pathways consistent with the systemic inflammation characterizing severe COVID-19 disease^5,6^. Indeed, evidence of a direct impact of miR-144-3p on inflammatory pathways has been documented both in LPS-induced cardiomyocyte injury^48^ and in macrophages^49,50^. Also relevant is miR-144-3p mediated protection against LPS-induced hyperpermeability of lung endothelial cells^51^. Additionally, miR-144-5p limits experimental abdominal aortic aneurysm formation attenuating M1-macrophage-associated inflammation^52^.

The cellular sources of plasma/serum miR-144 and cytokines are not known; therefore, a possible direct interaction between them may not be relevant in the COVID-19 context. The correlation between miR-144-3p and -5p plasma levels with those of IL-10 is weak and the correlation with the levels of EGF is not significant, suggesting the possibility that miR-144 and cytokines are biomarkers for different (although linked) aspects of the disease and corroborating the rationale for a combinatory use. Regardless of the existence of a direct interaction of miR-144 with IL-10 and EGF, we found that combined scores increased discriminatory power of disease severity and the prediction of mortality in COVID-19 patients, providing a robust stratification tool.

Our results should be interpreted in the context of some limitations. First, the size of our investigation did not allow to perform extensive analysis of all possible confounders. Second, only patients from European countries were recruited. As such, future studies in independent cohorts will need to further validate our results. Third, different measurement platforms and blood matrices were used, and a confirmatory study using standardized operative procedures is advised. Fourth, the saturation of critical care capacity during the first pandemic wave in Italy might have affected the composition of the COVID-19 patient groups. Fifth, the time elapsed between time of infection/symptoms onset and patient recruitment is not known and this may contribute to increased variability. Sixth, the enrolled patients were not matched for comorbidities that could be key risk factors for the onset and prognosis of COVID-19. Seventh, a potential impact on our results of hospital therapy, vaccination to SARS-CoV-2 and conditions and/or treatments that were not recorded cannot be excluded.

In conclusion, as summarized in supplementary Fig. 10, COVID-19 severity impacts on the miRNA profile of peripheral blood. Plasma miR-144, possibly in combination with IL-10 and EGF, emerges as a flexible noninvasive tool for risk-based stratification and mortality prediction of COVID-19 patients. Additional studies in larger patient groups displaying more ethnic diversification, along with functional approaches, will be necessary to validate these findings.

## Methods

### Ethics approval and consent to participate

Studies were performed in full compliance with the Declaration of Helsinki. The IRCCS Policlinico San Donato (PSD) experimental protocol was approved by the Institutional Ethics Committee of the San Raffaele Hospital (protocol number 75/INT/2020, of April 20 2020). The experimental protocol for the study conducted at the General University Hospital of Larissa (UGHL), University of Thessaly, was approved by the Ethics Committee of The University Hospital of Larissa, Thessaly, Greece (protocol number 28274, of July 6 2020). The Luxembourg Institute of Health (LIH) experimental protocol was approved by the National Research Ethics Committee of Luxembourg (study Number 202003/07, of April 2020). All the patients enrolled in these studies were asked for their informed consent as previously approved by the ethics committee of each center.

### Patient selection and sample collection

#### PSD patient group

The study conducted at PSD included 97 hospitalized patients aged 18 years or older, recruited during the period from March 2020 to April 2021 (Table 1) and 23 healthy controls who tested negative for the presence of antibodies against SARS-CoV-2 (64 ± 2.3 years old, range 60–69 years, 7 females and 16 males). Patients, all positively tested for SARS-CoV-2 by qPCR, were hospitalized at PSD and were categorized, according to severity of COVID-19 disease, in the following classes: (1) patients not requiring oxygen therapy, (2) patients requiring oxygen therapy, (3) patients requiring CPAP therapy and (4) patients admitted to ICU (Table 1). Participant data were collected at the time of hospital admission. According to internal Standard Operating Procedures, platelet-poor plasma and Peripheral Blood Mononuclear Cells (PBMC) samples were collected by BioCor Biobank at admission and then twice a week until patient discharge or death.

#### UGHL patient group

The study conducted at UGHL included 6 healthy controls that tested negative for SARS-CoV-2 in RT-qPCR assays (33 ± 7 years old, range 27–44 years, 3 females and 3 males) and 6 SARS-CoV-2-positive patients admitted directly to ICU (Supplementary Table 1) recruited during the period from April to July 2020. Serum samples were collected on admission.

#### LIH patient group

The study conducted at LIH included a subset of 76 participants of the Predi-COVID study^53^, recruited during the period from April to September 2020. This study enrolled non-hospitalized individuals aged 18 or older, positively tested for SARS-CoV-2 by qPCR. According to the NIH COVID-19 severity of illness scale^32^ participants were classified as mildly or moderately ill (Supplementary Table 2). Plasma samples and participant data were collected on study admission.

For all centers, peripheral blood samples were collected in ethylenediaminetetraacetic acid (EDTA) containing tubes and were centrifuged within two hours to separate plasma (1500 × *g* for 15 min at 4° C). To obtain platelet-poor plasma, samples were further centrifuged (14000 × *g* for 15 min at 4 °C). To obtain PBMCs, SepMate™ PBMC Isolation Tubes (STEMCELL TECHNOLOGIES) were employed, according to manufacturer’s instructions. To obtain serum, peripheral blood collected in serum-separator tubes was centrifuged at 1500 × *g* for 10 min. Serum, PBMCs and plasma samples were stored at − 80 °C.

### RNA isolation and RNA RT-qPCR quantification

Different protocols were employed for the three patient groups.

#### PSD patient group

The isolation of RNA, including miRNAs, from platelet-poor plasma was performed using NucleoSpin miRNA Plasma (MACHEREY–NAGEL), according to the manufacturer’s instructions. Considering the low amount of plasmatic RNAs and that UV–visible light-based quantification methods are inaccurate in plasma, same plasma volume was used for all patients. Isolation of total RNA from PBMCs was performed using TRIzol RNA Isolation Reagent (ThermoFisher Scientific) and NanoDrop One (ThermoFisher Scientific) was utilized to evaluate the RNA amount and purity. miRNA levels were analyzed using the TaqMan qPCR assay (Applied Biosystems) and quantified by the Step-One plus real-time PCR System (Applied Biosystems). Specific primers were provided by ThermoFisher Scientific. Expression values were normalized to U6 levels, as this endogenous normalizer has been shown to be less affected by inhibitors such as heparin than exogenous spike-ins^54^.

The levels of longer RNAs were measured by GoTaq® qPCR Master Mix according to the manufacturer’s protocol (Promega), normalizing to the averaged levels of RPL23 and UBC.

For all RNA species, relative expression was calculated using the comparative Ct (Delta Delta Ct) method^55^. Primer sequences are indicated in Supplementary materials.

#### UGHL patient group

Total RNA extraction from serum samples was performed with the In Vitro Diagnostic device for automated magnetic-bead-based extraction, magLEAD 12gC (Precision System Science Co.), following the manufacturer’s instructions, and stored at -80 °C.

#### LIH patient group

Total RNA, including miRNAs, was extracted from plasma samples using miRNeasy serum/plasma kit (Qiagen) according to the manufacturer’s instructions. Cel-miR-39 spike-in control was added during the extraction process to correct for extraction efficiency^56,57^. qPCR was performed utilizing specific primers and the miScript PCR System (Qiagen). For each qPCR plate, an internal control was used for inter-plate adjustment. Using cel-miR-39-3p as a normalization factor, expression levels for tested miRNAs were calculated as previously described^55,58^.

### Heparinase treatment

Before reverse transcription, the extracted RNA was treated with heparinase 1 from *Flavobacterium heparinum* (Sigma), according to the following protocol: 2 µL of each sample were combined with 1 µL heparinase (1 U/µL), 0.1 µL of RNase inhibitor (20 U/µL, Applied Biosystems), 0.3 µL of 10 × reverse transcription buffer (Applied Biosystems) and 0.2 µL of MgCl_2_ 25 mM (Promega), thoroughly mixed and incubated at 25 °C for 1 h. Samples were immediately used for reverse transcription. For comparison, the samples were also incubated under the same conditions without heparinase 1.

### Library preparation and miRNA sequence analysis

Protocols employed for miRNA sequence analysis of the PSD and UGHL patient groups are indicated in supplementary materials.

### Cytokine analysis

For Luminex assays, serum samples were gently vortexed and then centrifuged at 16,000 × *g* for 10 min at 4 °C immediately prior to testing with the Human XL Cytokine Premixed Kit (R&D Systems) which screens 45 analytes in the same specimen. Following the manufacturer’s instructions, 150 µl of serum were two-fold diluted with Calibrator Diluent RD6-65 and used in duplicate. Briefly, the fluorescent bead-based multiplexing immunoassay was run on Bio-Rad Bio-Plex 100 (Bio-Rad Laboratories), a dual-laser, flow-based sorting and detection platform. Magnetic microparticles precoated with cytokine-specific capture antibodies and embedded with fluorophores were reacted with serum samples. Next, a cocktail of biotinylated detection antibodies specific to the cytokines were bound to the cytokine-capture-antibody-microparticle complexes. The Streptavidin–phycoerythrin conjugate was then bound to the detection antibodies and excited by lasers in the Luminex analyzer to determine the concentration of each cytokine. Fluorescence data output was interpolated to standard curve by using the Bio Plex 6.0 software, and, after background subtraction and correction of duplicates for CV ≥ 10%, data of 31 cytokines passing all quality checks were used for further analysis. Multiple t-test corrected for multiple comparison with the Holm-Sidak was used to identify cytokines displaying significantly different levels in the analyzed groups (adjusted *p *< 0.05).

### TGF-β 1 ELISA assay

TGF-β 1 ELISA assay was performed using Human/Mouse TGF-β 1 uncoated ELISA kit (Invitrogen) according to the manufacturer’s instructions. The assay was performed in duplicate. Duplicates displaying an absorbance more than one standard deviation apart were not considered for the analysis.

### miRNA bioinformatics and pathway enrichment analysis

miR-144-3p and miR-144-5p predicted targets with conserved sites were identified using the Targetscan tool (release 8.0, http://www.targetscan.org/vert_80/)^34^Enriched pathway analysis of miRNA targets against Bioplanet ontologies/pathways was performed using EnrichR software^59,60^ (https://maayanlab.cloud/Enrichr/). DIANA-miTED database was used to explore miRNA tissue expression^47^.

### Statistical analysis

Continuous variables were expressed as mean ± standard error of the mean (SEM). For group-wise comparisons, Mann–Whitney test or unpaired t-test were used. Receiver operating characteristic (ROC) curve and area under the curve (AUC) were calculated to estimate the performance of a classification model at all classification thresholds. miR-144/cytokine combined scores were obtained averaging the ratios expressed in log_2_ scale for miR-144 and Interleukin-10 (IL-10) or miR-144 and Epidermal Growth Factor (EGF), calculated in the relevant patient groups. ANOVA test followed by Tukey’s post-hoc test was used to compare the means of more than two groups. All tests were performed two-sided and a *p *< 0.05 was considered as statistically significant. For statistical analysis, GraphPad Prism v.8.3.0 software (GraphPad Software Inc.) was used.

## Supplementary Information

Supplementary Information.

## Data Availability

miRNA profiling results are available on Gene Expression Omnibus, number GSE195898. Additional data that support the findings of this study, not publicly available due to privacy or ethical restrictions, can be obtained on request from the corresponding authors.

## Abbreviations

ANOVA: Analysis of variance
AUC: Area under the ROC curve
CPAP: Continuous positive airway pressure
CPM: Count per million
EGF: Epidermal growth factor
ICU: Intensive care unit
IL-10: Interleukin-10
LIH: Luxembourg Institute of Health
miRNA: MicroRNA
PBMCs: Peripheral blood mononuclear cells
PSD: IRCCS Policlinico San Donato
ROC: Receiver operating characteristic
SARS-CoV-2: Severe acute respiratory syndrome coronavirus 2
SEM: Standard error of the mean
UGHL: General University Hospital of Larissa
