# Supplementary material for: Association of miR-144 levels in the peripheral blood with COVID-19 severity and mortality
Source: Sci Rep. 2022 Nov 21;12:20048. doi: 10.1038/s41598-022-23922-2 (PMC9681736; doi:10.1038/s41598-022-23922-2)
Supplement: Supplementary file 1 — Supplementary Information. [file 41598_2022_23922_MOESM1_ESM.pdf]

Title: Association of miR-144 levels in the peripheral blood with COVID-19 severity and mortality

Alisia Madè[1], Simona Greco[1], Melanie Vausort[2], Marios Miliotis[3,4], Eric Schordan[5], Shounak Baksi[2], Lu Zhang[2], Ekaterina Baryshnikova[6], Marco Ranucci[6], Rosanna Cardani[7], Guy Fagherazzi[8], Markus Ollert[9,10], Spyros Tastsoglou[3,4], Giannis Vatsellas[11], Artemis Hatzigeorgiou[3,4], Hüseyin Firat[5], Yvan Devaux[2*], Fabio Martelli[1*]

1) Molecular Cardiology Laboratory, IRCCS Policlinico San Donato, via Morandi 30, 20097, San Donato Milanese, Milan, Italy.
2) Cardiovascular Research Unit, Department of Precision Health, Luxembourg Institute of Health, L-1445 Strassen, Luxembourg.
3) Hellenic Pasteur Institute, 11521 Athens, Greece.
4) DIANA-Lab, Department of Computer Science and Biomedical Informatics, University of Thessaly, 35131 Lamia, Greece.
5) Firalis SA, 35 Rue du Fort, 68330 Huningue, France.
6) Department of Cardiovascular Anesthesia and ICU, IRCCS Policlinico San Donato, via Morandi 30, 20097, San Donato Milanese, Milan, Italy.
7) BioCor Biobank, UOC SMEL-1 of Clinical Pathology, Department of Pathology and Laboratory Medicine, IRCCS-Policlinico San Donato, via Morandi 30, 20097, San Donato Milanese Milan, Italy.
8) Deep Digital Phenotyping Research Unit. Department of Precision Health. Luxembourg Institute of Health, 1 A-B rue Thomas Edison, L-1445 Strassen, Luxembourg.
9) Department of Infection and Immunity. Luxembourg Institute of Health, 29, Rue Henri Koch, L-4354 Esch-sur-Alzette, Luxembourg.
10) Department of Dermatology and Allergy Center, Odense Research Center for Anaphylaxis (ORCA), University of Southern Denmark, Odense, 5000 C, Denmark.

11) Greek Genome Center, Biomedical Research Foundation, Academy of Athens, Athens 11527, Greece.

*Corresponding authors:

Fabio Martelli: Molecular Cardiology Laboratory, IRCCS Policlinico San Donato, via Morandi 30, 20097, San Donato Milanese, Milan, Italy. E-mail address: fabio.martelli@grupposandonato.it. Phone number: +39 0226437762

Yvan Devaux: Cardiovascular Research Unit, Department of Precision Health, Luxembourg Institute of Health, L-1445 Strassen, Luxembourg. E-mail address: yvan.devaux@lih.lu. Phone number: +352 26970-303

SUPPLEMENTARY METHODS

Primer sequences

IL-10 FW: TCTCCGAGATGCCTTCAGCAGA; IL-10 REV: TCAGACAAGGCTTGGCAACCCA

EGF FW: TGCGATGCCAAGCAGTCTGTGA; EGF REV: GCATAGCCCAATCTGAGAACCAC

pri-miR-144 FW: TGCCTTGTTTGAGCTGGAGT; pri-miR-144 REV:CTCTGCTCAGCCTGTCACAA

UBC FW: GATCGCTGTGATCGTCACTTGACAA; UBC REV: AGTCAGACAGGGTGCGCCCA

RPL23 FW: TCCGGATTTCCTTGGGTCTT; RPL23 REV: TGTTCAGCCGTCCCTTGATC

Library preparation and miRNA sequence analysis

PSD patient group. Profiling of 2084 miRNAs was performed at FIRALIS using HTG EdgeSeq miRNA Whole Transcriptome targeted sequencing kit (HTG WTA, HTG Molecular). Samples were prepared according to the manufacturer's instructions. Barcoding was performed using Hemo KlenTaq (MO332S, NEB) enzyme. For each sample, they were mixed 2.4 µl of Hemo KlenTaq, 0.6

µl of dNTPs (10 nM, N0447S, NEB), 6 µl of OneTaq PCR GC Buffer 5X (B9023S, NEB), 3 µl of Forward and Reverse Primers (HTG WTA, HTG Molecular), 3 µl of sample and 12 µl of H_2O. PCR was performed on BioRad T100 Thermocycler using the following cycling profile: 95°C for 4 min, followed by 16 cycles at 95°C for 15 sec, 55°C for 45 sec and 68°C for 45 sec. The protocol was concluded with 68°C for 10 min. Libraries were cleaned up from primer excess using Agentcour AMPure XP beads (A63880, Beckman Coulter). After library QC and concentration evaluation, samples were pooled in order to generate a pooled library at 4 nM which was loaded into NextSeq High Output v2 75 cycles kit and sequenced (Gene Expression Omnibus number GSE195898). The sequencing depth was ≥ 1 million reads per sample at 1 x 50 bp. Sequencing data were first analyzed and checked using the Q30 metric. Data reconstruction and analysis of FASTQ files were obtained by the HTG Parser software. Before data normalization, a negative control (ANT) QC was performed on parsed raw data. Samples with high number of reads in negative control (over 150 count per million (CPM)) were flagged as QC failure and removed from the analysis. After background subtraction, all negative values were set to 0. We performed quantile normalization and paired test using R package limma (35) for surviving and nonsurviving patients, respectively. Only the miRNAs showing ≥ 50 CPM in at least half of the samples of one group were considered as detected. Results were sorted by ascending p-values and the data of top 10 miRNAs were employed to generate a heatmap using R package pheatmap (https://cran.r-project.org/web/packages/pheatmap/index.html).

UGHL patient group. sRNA-Seq libraries were prepared using QIAseq miRNA Library Kit (Qiagen) with its standard 3' adapter (AACTGTAGGCACCATCAAT) followed by 12-mer Unique Molecular Indices (UMIs). NGS Sequencing was performed at Greek Genome Center – BRFAA on a NextSeq 550 System (Illumina), producing a sequencing depth of 7.6 million 75bp single-end reads on average. UMIs and adapters were detected and removed with umi-tools 1.0.1. Quality in raw and preprocessed sequencing files was controlled using FastQC Suite (www.bioinformatics.babraham.ac.uk/projects/fastqc/) and Minion v.15-065 from the EBI Kraken tools (https://www.ebi.ac.uk/research/enright/software/kraken) to determine overrepresentation of adapters and sequences of non-host origin. Briefly, sequences found overrepresented >10% were

queried against *Homo sapiens* Nucleotide collection with BLASTn and against mature miRNAs in miRBase (https://www.mirbase.org/). Removal of potential contaminants and quality trimming was performed with Trim Galore 0.4.4 (www.bioinformatics.babraham.ac.uk/projects/trim_galore/). A mean of 93% of short reads were mapped to the human genome, out of which 60% on average were assigned to miRNAs. Prior to performing statistical analysis, lowly abundant miRNAs were filtered out by retaining only those with >10 CPM in at least 6 samples. Raw counts were normalized for each library using the total counts quantified by Manatee tool (36). For the abundance analysis of the sequenced data, Manatee tool was used at default settings. Differential expression analysis was carried out using the R programming language (version 3.6.3) by employing the voom function from R package limma (version 3.42.2) (35,37). Moderated statistics and log-odds were computed using the treat function from limma. False discoveries were controlled by adjusting p-values for multiple comparisons using the Benjamini-Hochberg procedure (threshold set at 0.05).

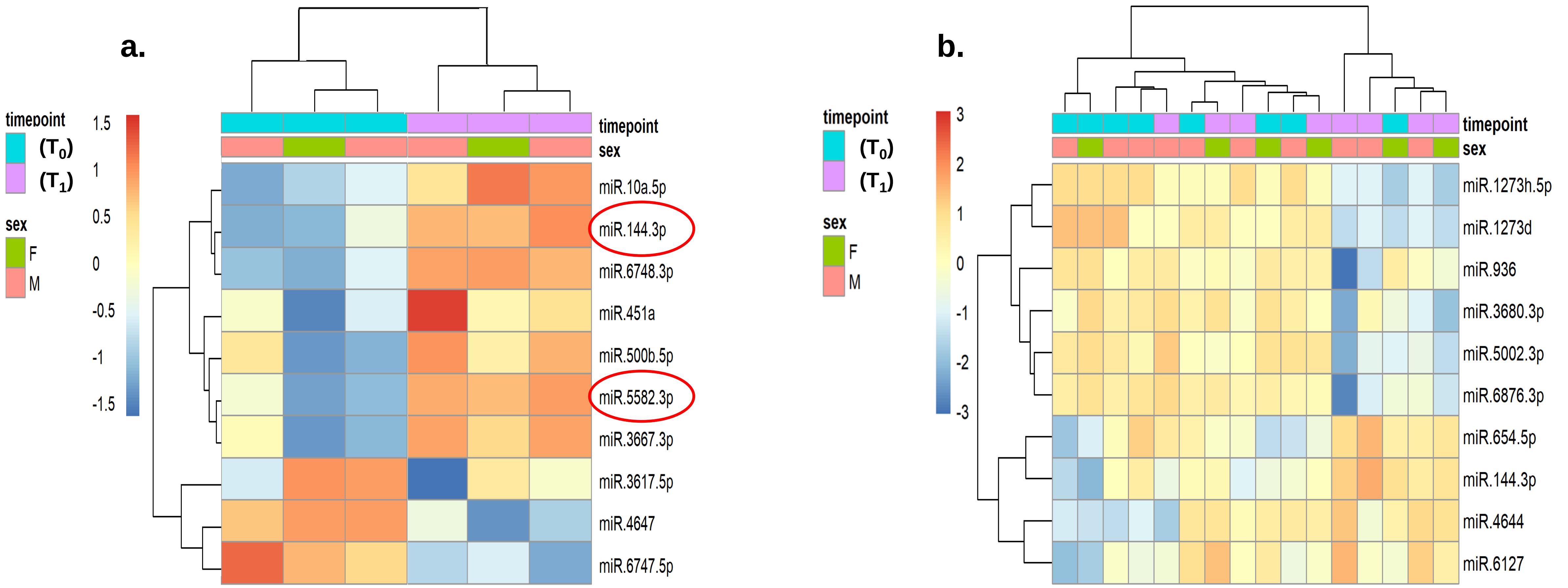

Supplementary Figure 1. Top differentially expressed miRNAs comparing T_1 and T_0 samplings in surviving and nonsurviving COVID-19 patients. *The heatmaps indicate, ranked by differential expression values comparing T_1 and T_0 samplings, the top 10 miRNAs in (a) surviving (n= 3) and (b) nonsurviving (n= 8) COVID-19 patients. Red circles indicate differentially expressed miRNAs with a p-value < 0.001. Unsupervised hierarchical clustering discriminates T_0 from T_1 timepoints only in surviving patients.*

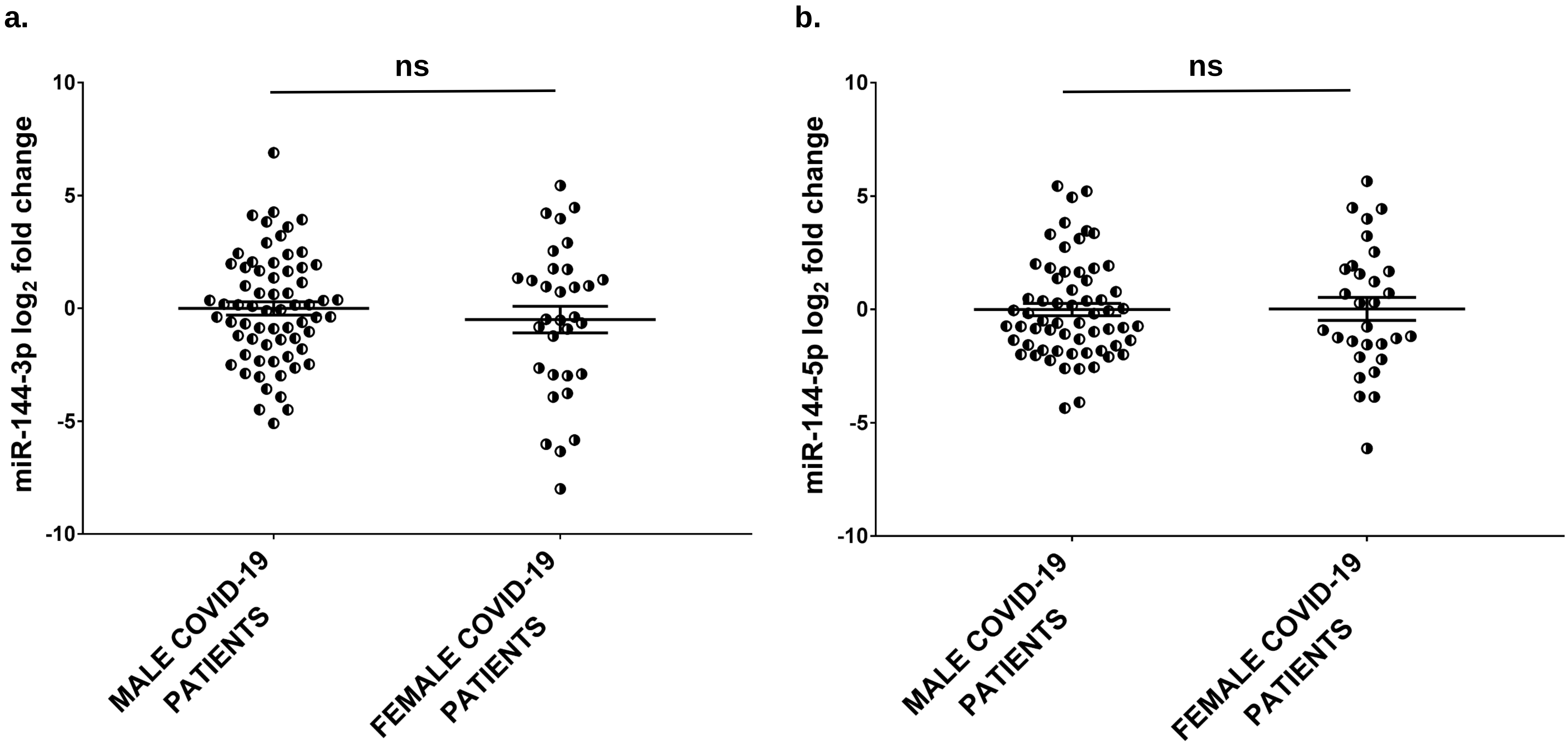

Supplementary Figure 2. No significant difference in miR-144 levels between male and female hospitalized COVID-19 patients. *miR-144-3p (a) and miR-144-5p (b) plasma levels were measured by qPCR. Values are expressed as log_2 fold change and shown as dot-plots indicating mean ± SEM. Unpaired t-test (two groups) was used for statistical comparison. Male COVID-19 patients n= 65-61; female COVID-19 patients n= 32-30.*

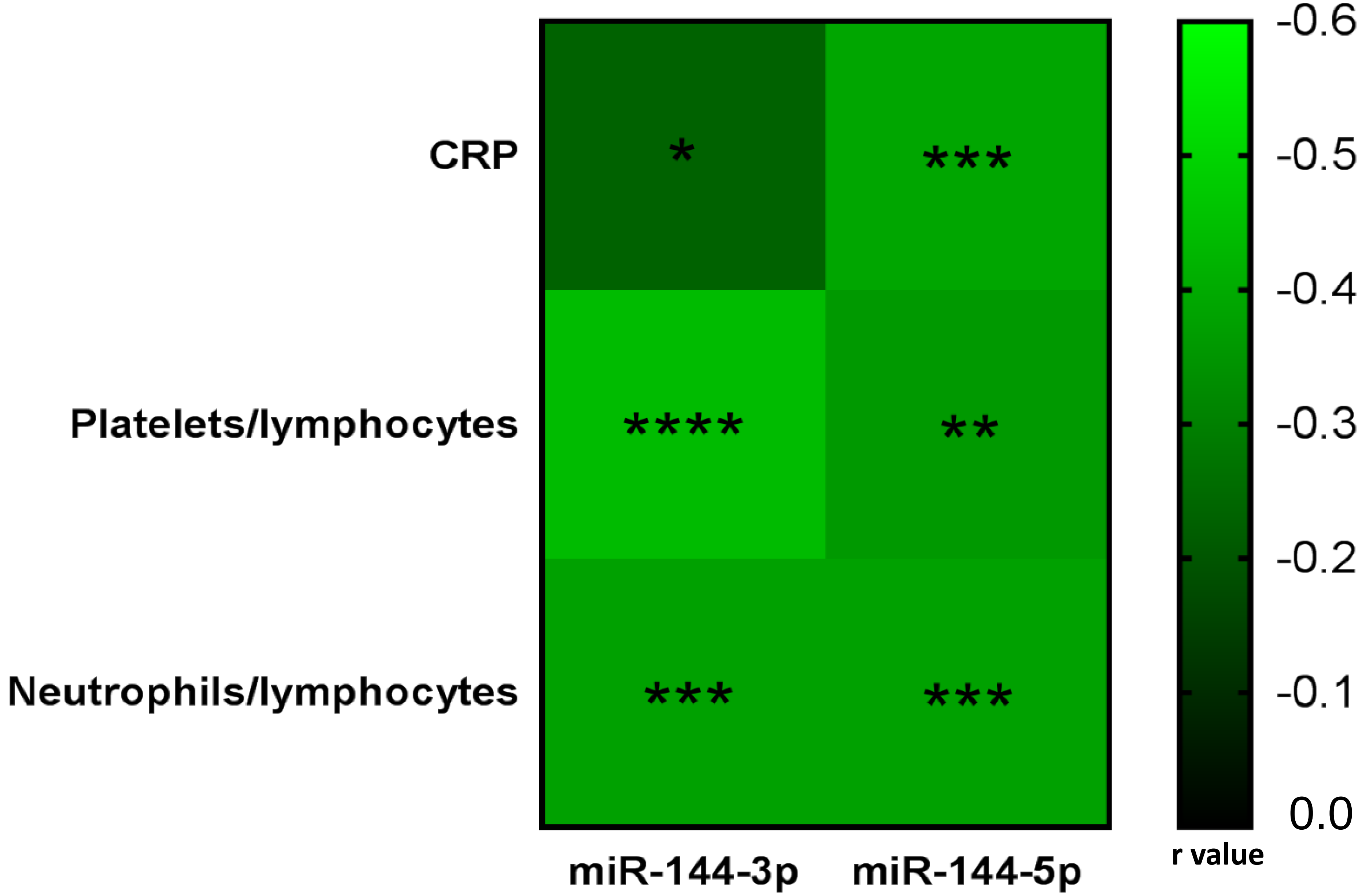

Supplementary Figure 3. Heat-map displaying the correlations of miR-144-3p and -5p levels with clinically relevant parameters in hospitalized COVID-19 patients recruited at PSD. *Brighter green color indicates stronger negative correlation (Spearman's r correlation coefficient). Stars indicate statistical significance (*p< 0.05; **p≤ 0.01;***p≤ 0.005; ****p≤ 0.0005; n= 55-62).*

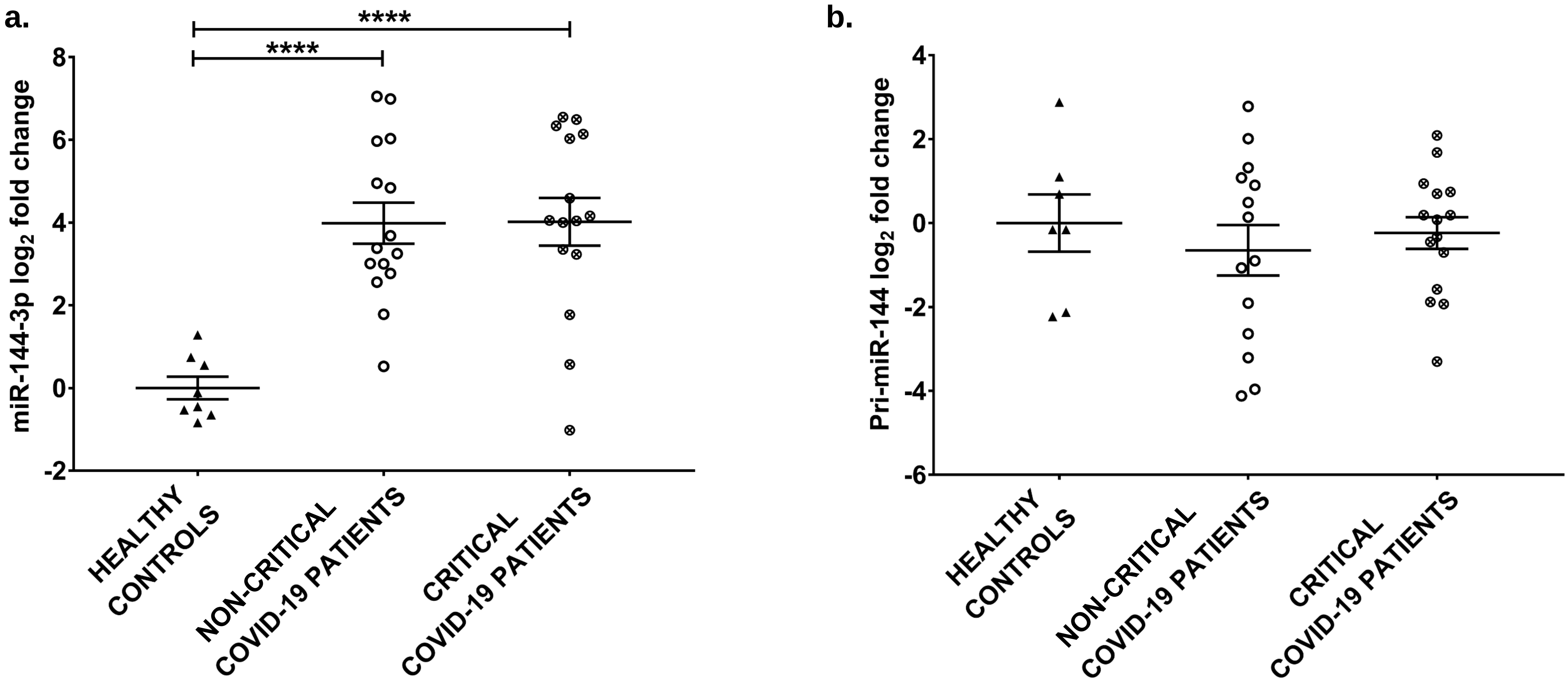

Supplementary Figure 4. Mature miR-144-3p levels in PBMCs are increased in hospitalized COVID-19 patients. *The levels of mature (a) and precursor (b) miR-144-3p were measured by qPCR in the PBMCs of hospitalized patients at PSD and in healthy controls. Values are expressed as log_2 fold change compared to controls and shown as dot-plots indicating mean ± SEM. Expression levels of mature miR-144-3p were increased in both non-critical and critical COVID-19 patients compared to healthy controls; conversely, the levels of precursor miR-144 were not affected significantly. ANOVA test, followed by Tukey's post-hoc test, was performed for statistical comparison. In panel (a), Controls n= 8; non-critical patients n= 15; critical patients n= 15; **** p≤ 0.0001. In panel (b), Controls n= 7; non-critical patients n= 14; critical patients n= 15.*

Supplementary Table 1. Characteristics of the UGHL group of hospitalized COVID-19 patients.

		UGHL COVID-19 PATIENTS (n = 6)
**Age, median (range)**		67 (48-71)
**Gender, male, n (%)**		3 (50)
**Medical history/comorbidities, n (%)**		
**Smoking**	**Current smoker**	3 (50)
	**Former smoker**	1 (17)
**Hypertension**		5 (83)
**Diabetes**		3 (50)
**Obesity**		2 (33)
**Cardiovascular diseases**		2 (33)
**Chronic obstructive pulmonary disease**		2 (33)
**Asthma**		1 (17)
**Cancer**		2 (33)
**Chronic kidney disorders**		0 (0)
**Other disorders**		6 (100)

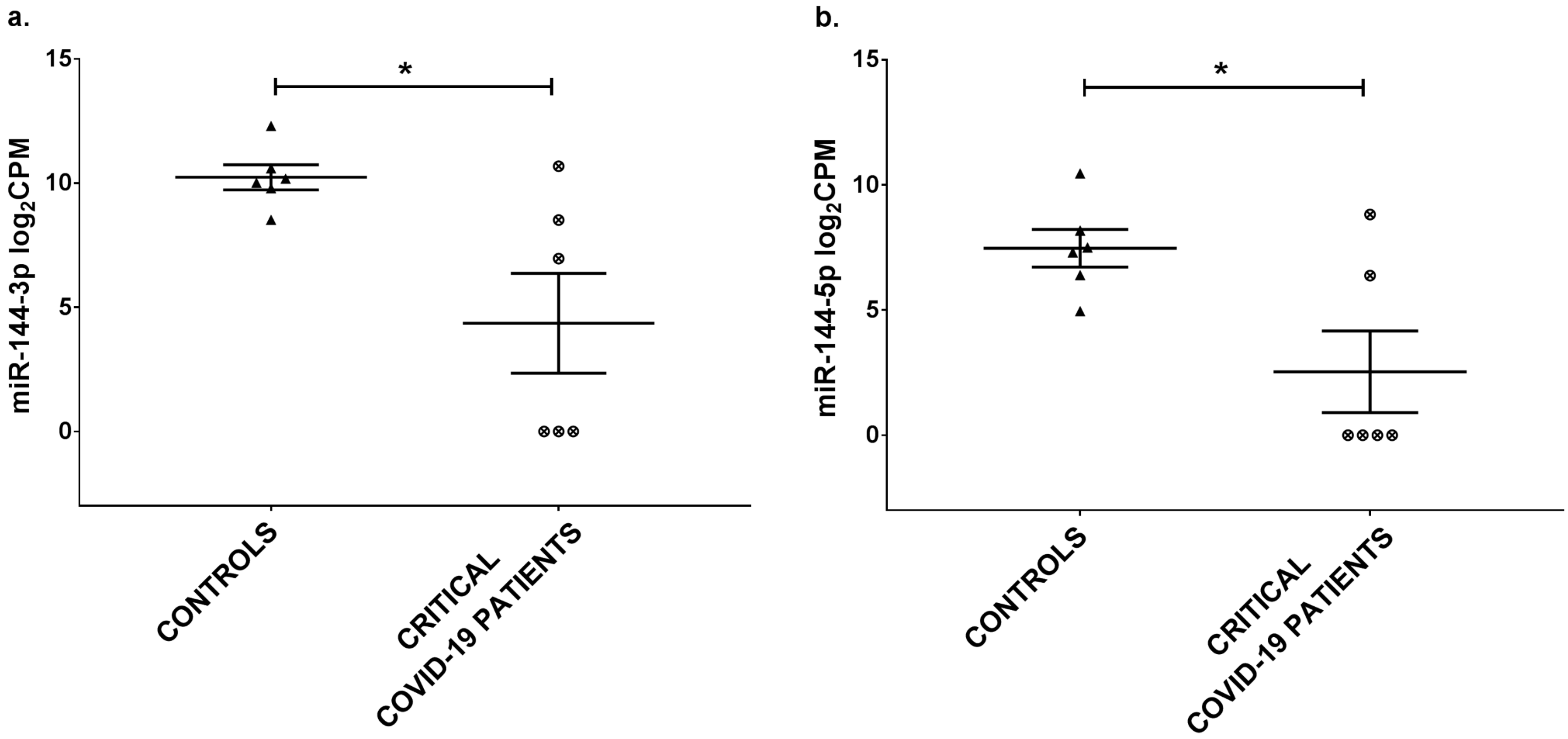

Supplementary Figure 5. miR-144 levels in controls and COVID-19 patients hospitalized at UGHL. *Critical patients admitted at ICU (n= 6) were compared to 6 matched healthy subjects (n= 6). Plasma levels of miR-144-3p (a) and miR-144-5p (b) were measured by RNA-sequencing. Values are expressed as* log_2 *CPM mapped reads and shown as dot- plots indicating mean ± SEM. Mann-Whitney test (two groups) was performed for statistical comparison (** $p< 0.05$*).*

Supplementary Table 2. Characteristics of the LIH group of non-hospitalized COVID-19 patients. *Patients were classified according to NIH COVID-19 severity of illness scale.*

		LIH COVID-19 PATIENTS		
		**All (n = 76)**	**Mild illness (n = 59)**	**Moderate illness (n = 17)**
**Age, median (range)**		36 (19-74)	36 (19-74)	36 (23-57)
**Gender, male, n (%)**		44 (58)	35 (59)	9 (53)
**Body Index mass, median (range)**		25.6 (17.3-39.9)	25.2 (17.3-37.6)	26.9 (20.3-39.9)
**Medical history/comorbidities, n (%)**				
**Smoking**	**Current smoker**	13 (17)	10 (17)	3 (8)
	**Former smoker**	15 (20)	13 (22)	2 (12)
**Hypertension**		6 (8)	4 (7)	2 (12)
**Diabetes**		4 (5)	4 (7)	0 (0)
**Obesity**		4 (5)	2 (3)	2 (12)
**Chronic cardiac disorders**		4 (5)	2 (3)	2 (12)
**Chronic pulmonary disorders (except asthma)**		0 (0)	0 (0)	0 (0)
**Asthma**		7 (9)	5 (8)	2 (12)
**Chronic obstructive pulmonary disease**		1 (1)	1 (2)	0 (0)
**Cancer**		2 (3)	1 (2)	1 (6)
**Neurological disorders**		0 (0)	0 (0)	0 (0)
**Chronic kidney disorders**		0 (0)	0 (0)	0 (0)
**Dialysis**		0 (0)	0 (0)	0 (0)
**Moderate or severe liver disorders**		1 (1)	1 (2)	0 (0)
**Chronic blood disorders**		4 (5)	2 (3)	2 (12)
**AIDS**		0 (0)	0 (0)	0 (0)
**Rheumatological disorders**		1 (1)	1 (2)	0 (0)
**Other disorders**		16 (21)	12 (20)	4 (24)

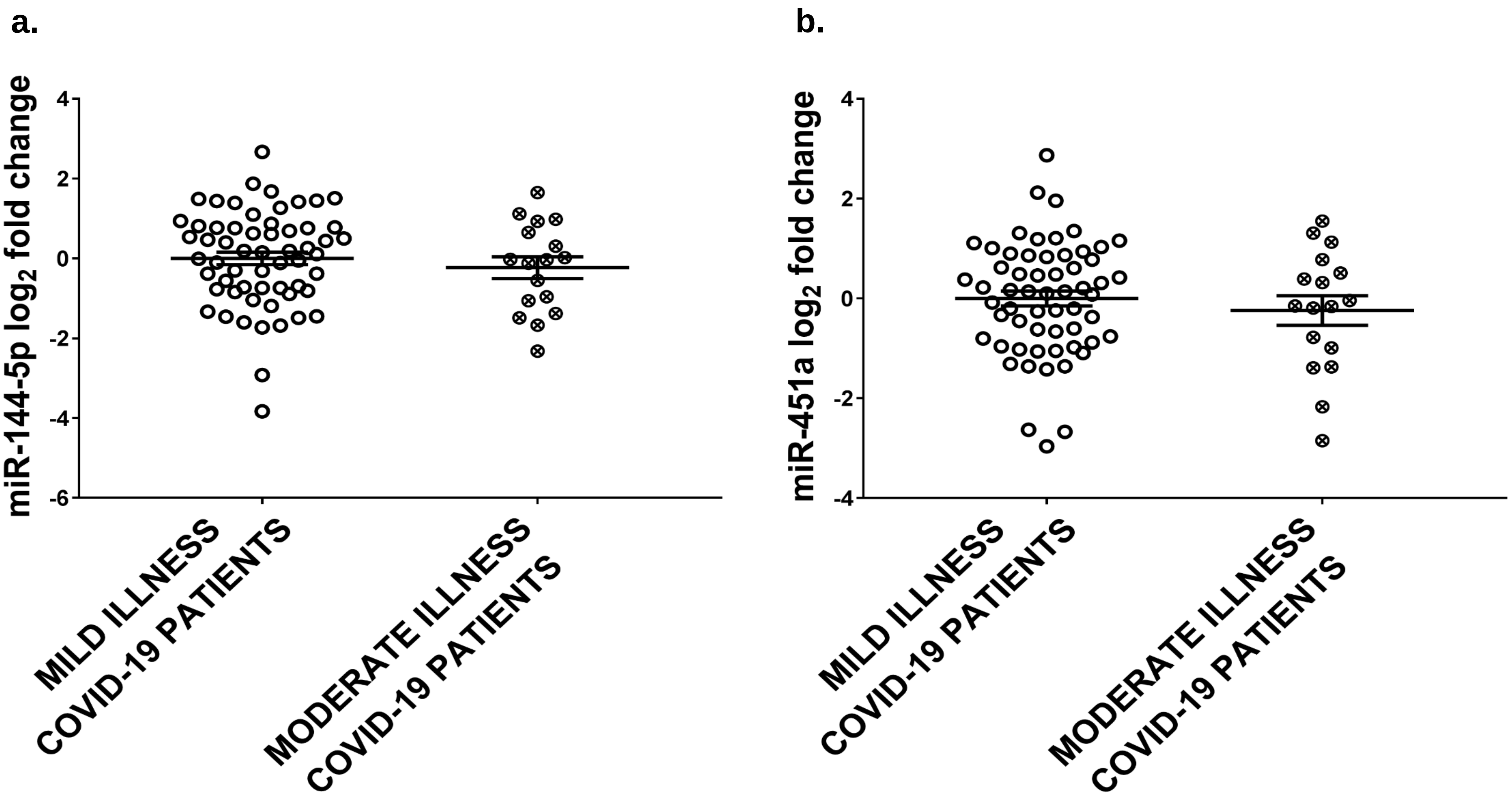

Supplementary Figure 6. No significant difference in miR-144-5p and miR-451a levels between mild and moderate non-hospitalized COVID-19 patients recruited at LIH. *miR-144-5p (a) and miR-451a (b) plasma levels were measured by qPCR. Values are expressed as* log_2 *fold change compared to mild illness patients and shown as dot-plots indicating mean ± SEM. Unpaired t-test (two groups) was used for statistical comparison and no significant difference was observed. Mild illness patients n= 59; moderate illness patients n= 17.*

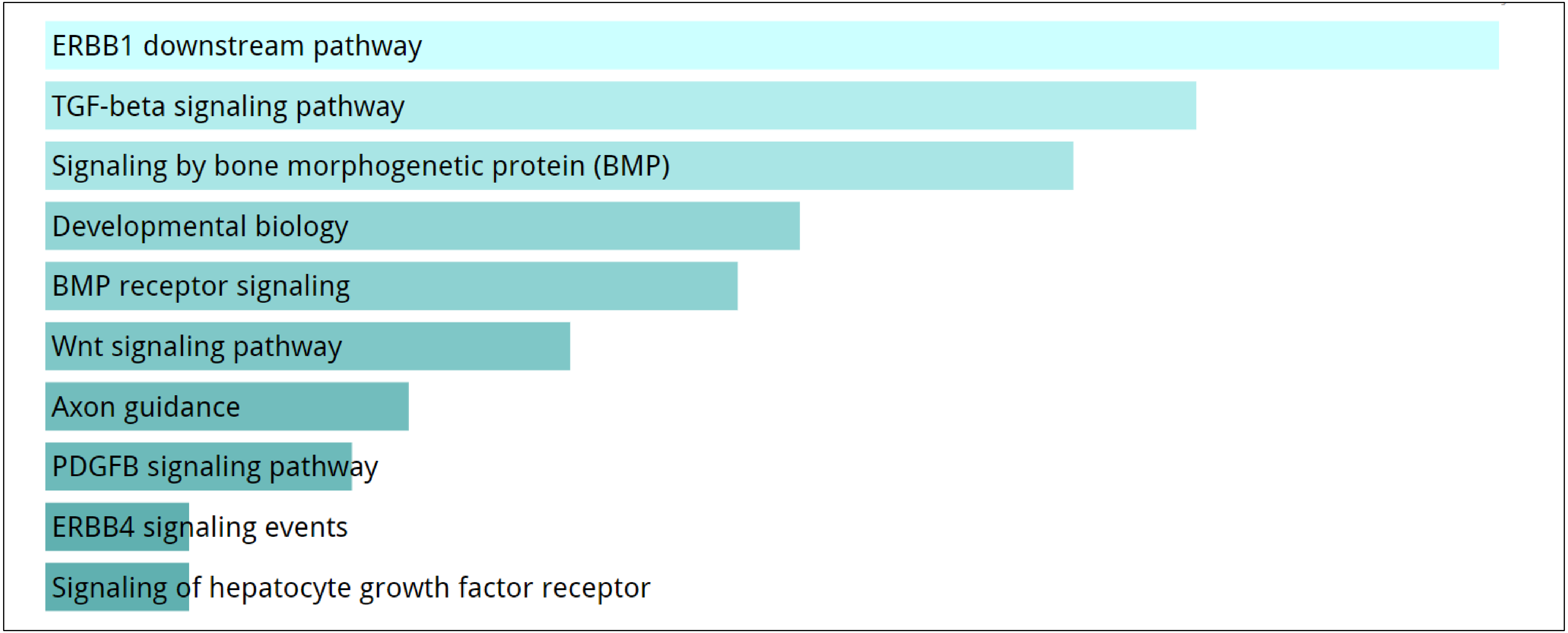

Supplementary Figure 7. Pathway enrichment analysis of miR-144-3p and -5p predicted targets. *Targets were predicted using the Targetscan (release 8.0, https://www.targetscan.org/vert_80/) and their analysis was performed using EnrichR tool (March, 29th 2021, https://maayanlab.cloud/Enrichr/). The length of the bar represents the significance of each term. In addition, the brighter the color, the more significant that term is. Bars are sorted by p value ranking.*

Supplementary Table 3. Characteristics of the COVID-19 patients hospitalized at PSD used for serum cytokines analysis.

		COVID-19 PATIENTS				
		All (n = 62)	Severity 1 † (n=3)	Severity 2 ‡ (n=14)	Severity 3 § (n=18)	Severity 4 ¶ (n=27)
**Age, median (range)**		68 (26-86)	67 (58-78)	63 (26-85)	65 (50-85)	70 (48-86)
**Gender, male, n (%)**		38 (61)	1 (33)	8 (57)	11 (61)	18 (67)
**Outcome, dead patients (%)**		20 (32)	0 (0)	1 (7)	6 (33)	13 (48)
**Days of hospitalization, median**		11	5	7	15	8
**Medical history/comorbidities, n (%)**						
**Smoking**	**Current smoker**	1 (2)	0 (0)	0 (0)	1 (6)	0 (0)
	**Former smoker**	7 (11)	0 (0)	2 (14)	1 (6)	4 (15)
**Hypertension**		37 (60)	2 (67)	7 (50)	13 (72)	15 (56)
**Diabetes**		10 (16)	1 (33)	1 (7)	2 (11)	6 (22)
**Obesit**		14 (23)	1 (33)	2 (14)	2 (11)	9 (33)
**Ischemic cardiomyopathy**		13 (21)	0 (0)	3 (21)	5 (28)	5 (18)
**Chronic heart failure**		5 (8)	0 (0)	1 (7)	4 (22)	0 (0)
**Atrial fibrillation**		4 (6)	0 (0)	2 (14)	2 (11)	0 (0)
**Left ventricular dysfunction**		2 (3)	0 (0)	1 (7)	1 (6)	0 (0)
**Congenital heart diseases**		0 (0)	0 (0)	0 (0)	0 (0)	0 (0)
**Chronic obstructive pulmonary disease**		4 (6)	0 (0)	1 (7)	0 (0)	3 (11)
**Asthma**		3 (5)	0 (0)	2 (14)	0 (0)	1 (4)
**Cancer**		3 (5)	0 (0)	1 (7)	2 (11)	0 (0)
**Pre-existing stroke**		1 (2)	0 (0)	0 (0)	0 (0)	1 (4)
**Chronic neurological disorders**		2 (3)	0 (0)	1 (7)	1 (6)	0 (0)
**Chronic kidney disorders**		2 (3)	0 (0)	1 (7)	1 (6)	0 (0)
**Liver disorders**		0 (0)	0 (0)	0 (0)	0 (0)	0 (0)
**Chronic gut inflammation**		0 (0)	0 (0)	0 (0)	0 (0)	0 (0)
**Other disorders**		27 (44)	3 (100)	7 (50)	9 (50)	8 (30)
**Hospital therapy, n (%)**						
**Antiviral drug**		16 (26)	0 (0)	1 (7)	1 (6)	14 (52)
**Cortisone therapy**		5 (82)	2 (67)	12 (86)	14 (78)	23 (85)
**Immunosuppressive therapy**		2 (3)	0 (0)	0 (0)	1 (6)	1 (4)
**Muscle relaxant therapy**		25 (40)	0 (0)	0 (0)	0 (0)	25 (93)
**Amine therapy**		14 (23)	0 (0)	0 (0)	0 (0)	14 (52)
**Sedative**		27 (44)	0 (0)	1 (7)	1 (6)	25 (93)
**Hydroxychloroquine/chloroquine**		23 (37)	1 (33)	2 (14)	7 (39)	13 (48)
**Antibiotic therapy**		57 (92)	3 (100)	12 (86)	17 (94)	25 (93)
**Therapy with NSAID**		5 (8)	0 (0)	1 (7)	1 (6)	3 (11)
**Antifungal therapy**		3 (5)	0 (0)	1 (7)	0 (0)	2 (7)
**Anticoagulant therapy**		59 (95)	3 (100)	13 (93)	17 (94)	26 (96)

†*Severity 1: patients not requiring oxygen therapy;* ‡ *Severity 2: patients requiring oxygen therapy;* § *Severity 3: patients requiring CPAP therapy;* ¶ *Severity 4: ICU-admitted patients with endotracheal intubation.*

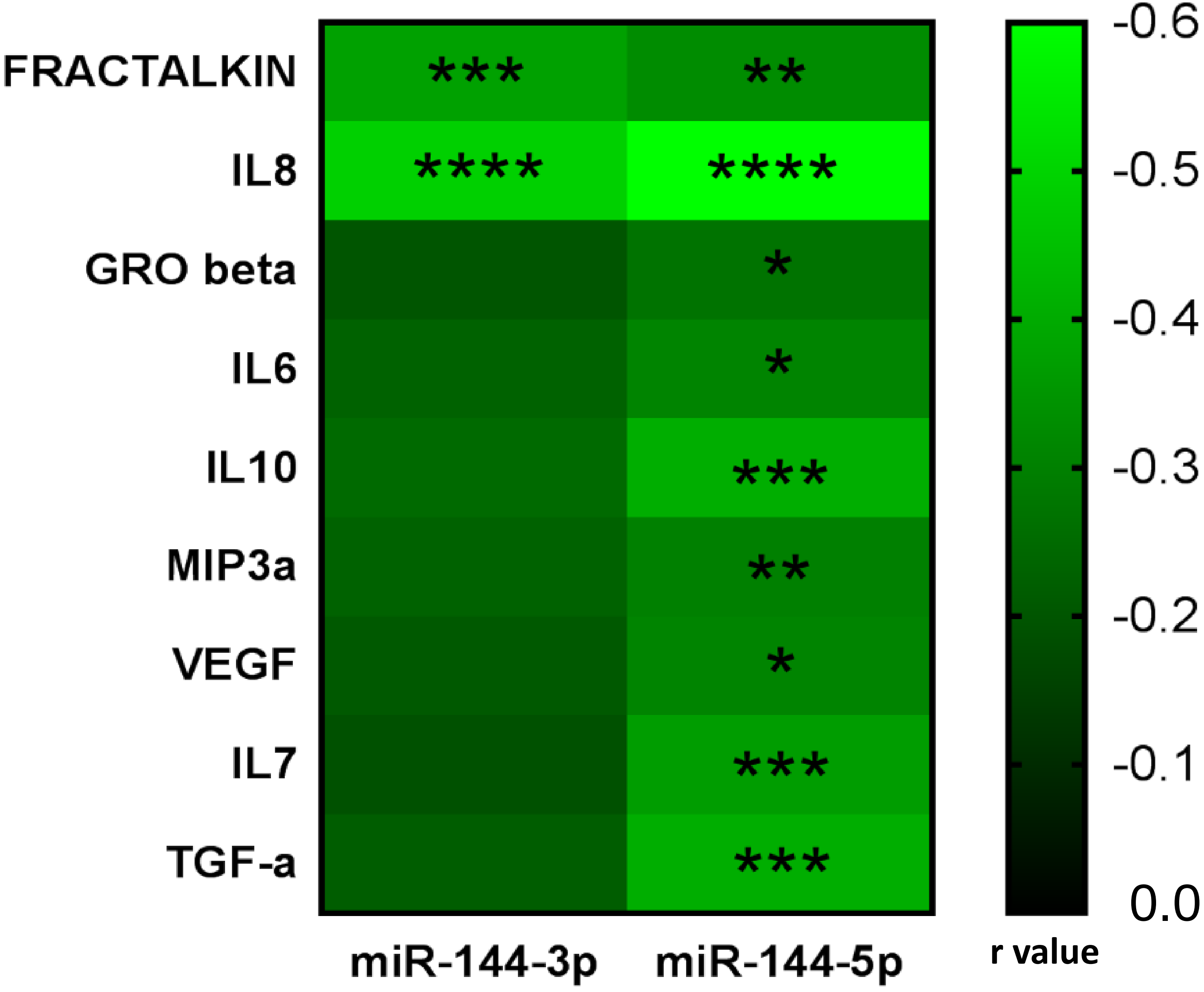

Supplementary Figure 8. Heat-map displaying the correlations of miR-144-3p and -5p levels with inflammatory cytokines in hospitalized COVID-19 patients recruited at PSD. *Brighter green color indicates stronger negative correlation (Spearman's r correlation coefficient). Stars indicate statistical significance (* p< 0.05; ** p≤ 0.01;*** p≤ 0.005; **** p≤ 0.0005; n= 53-62).*

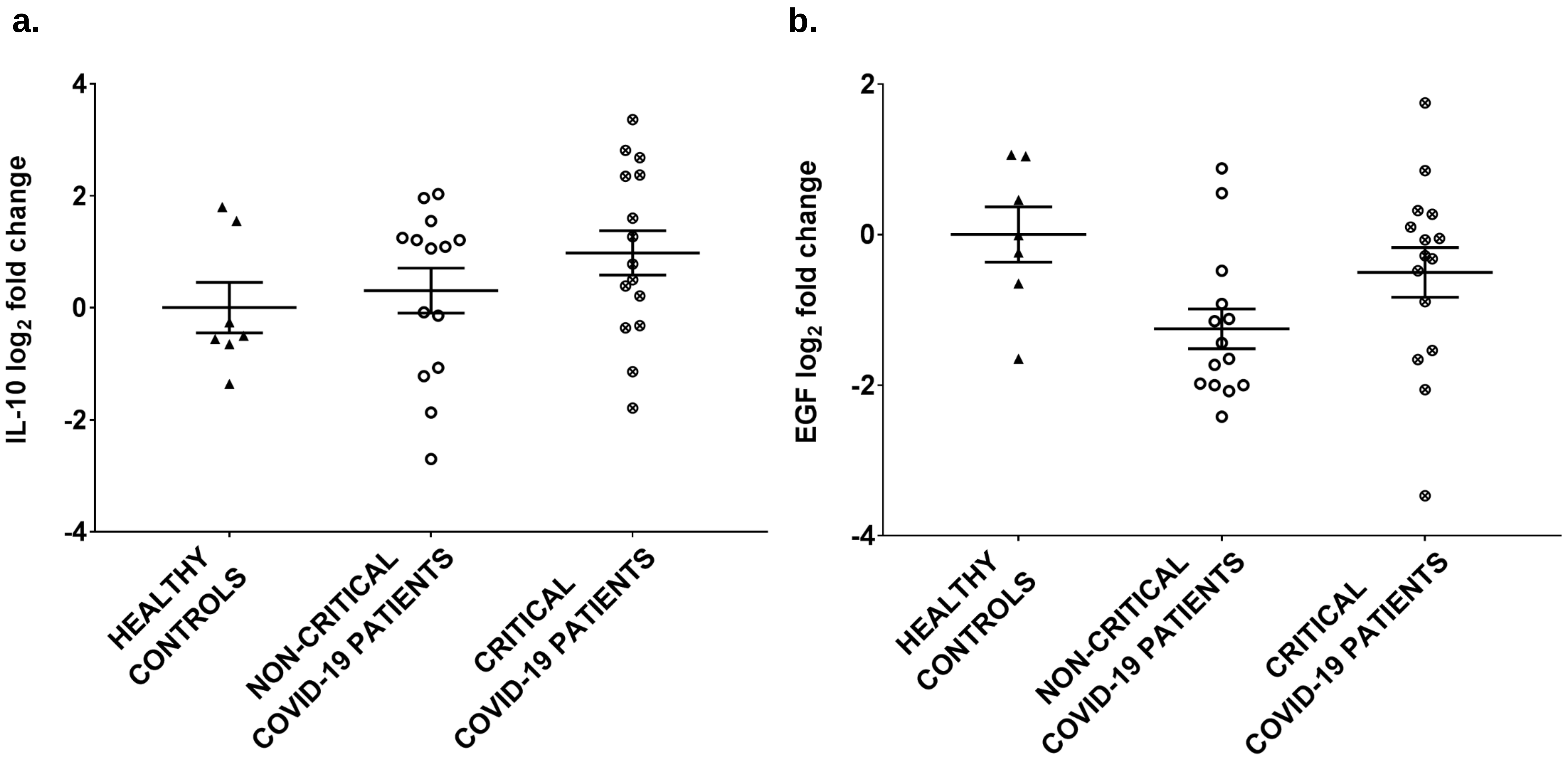

Supplementary Figure 9. No significant difference in IL-10 and EGF transcript levels between PBMCs of hospitalized COVID-19 patients and healthy controls. *PBMC levels of IL-10 (a) and EGF (b) mRNAs were measured by qPCR. Values are expressed as log2 fold change compared to healthy controls and shown as dot-plots indicating mean ± SEM. ANOVA test, followed by Tukey's post-hoc test, was performed for statistical comparison and no significant differences were observed. Controls n= 7; non-critical patients n= 14; critical patients n= 15.*

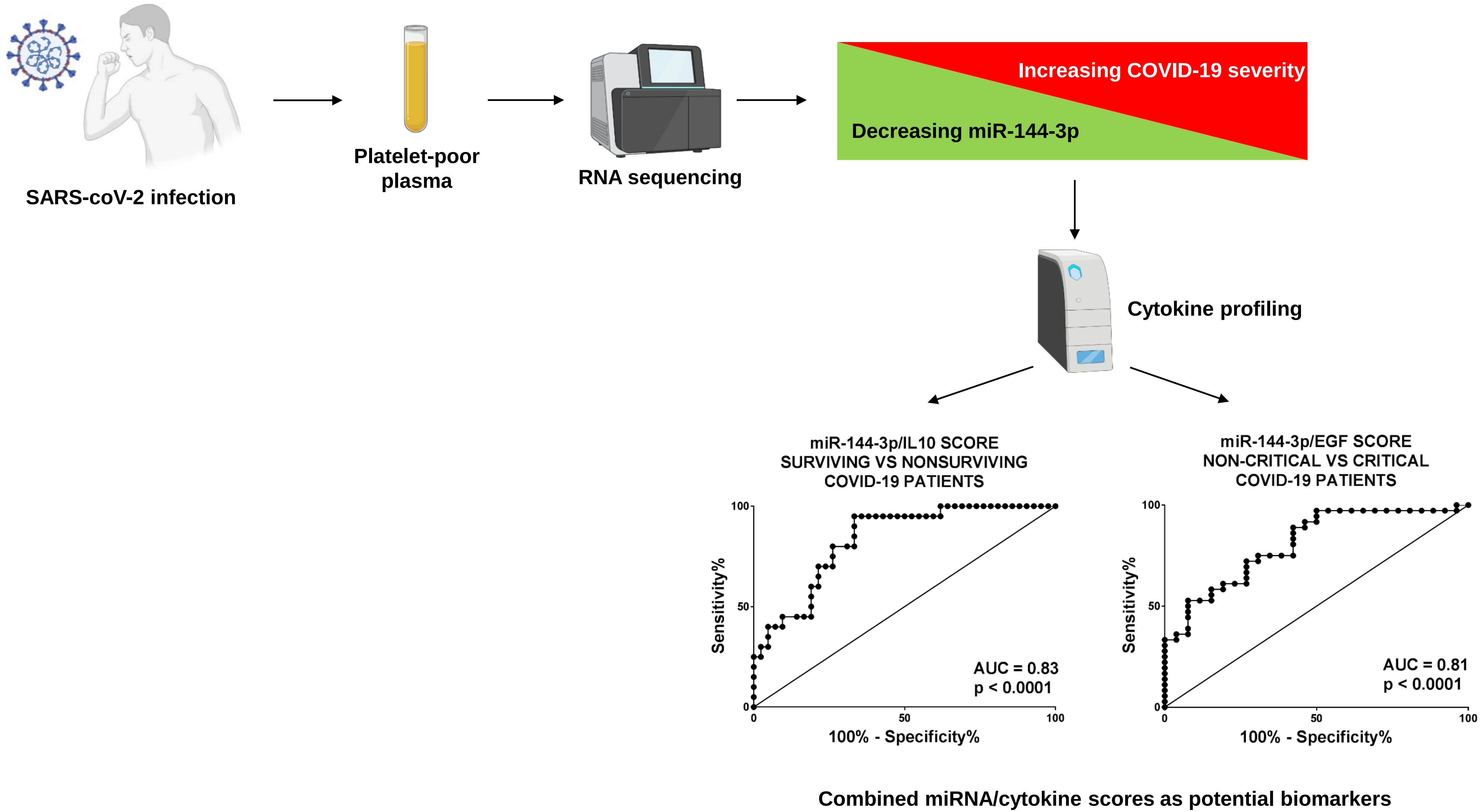

Supplementary Figure 10. *Schematic representation of the main findings of the study, indicating the association of miR-144 levels in the peripheral blood with COVID-19 severity and its potential use as biomarker combined with IL10 and EGF. Figure was partially generated by using BioRender 2022 (https://biorender.com/).*
